# Quantitative estimate of cognitive resilience and its medical and genetic associations

**DOI:** 10.1186/s13195-023-01329-z

**Published:** 2023-11-06

**Authors:** Thanaphong Phongpreecha, Dana Godrich, Eloise Berson, Camilo Espinosa, Yeasul Kim, Brenna Cholerton, Alan L. Chang, Samson Mataraso, Syed A. Bukhari, Amalia Perna, Koya Yakabi, Kathleen S. Montine, Kathleen L. Poston, Elizabeth Mormino, Lon White, Gary Beecham, Nima Aghaeepour, Thomas J. Montine

**Affiliations:** 1 Department of Pathology, Stanford University, Stanford, CA USA; 2 Department of Anesthesiology, Perioperative, and Pain Medicine, Stanford University, Stanford, CA USA; 3 Department of Biomedical Data Science, Stanford University, 300 Pasteur Dr Rm L216, Stanford, CA 94305 USA; 4 Dr. John T. Macdonald Foundation Department of Human Genetics, University of Miami, Miami, FL USA; 5 Department of Pediatrics, Stanford University, Stanford, CA USA; 6 Department of Neurology Neurological Sciences, Stanford University, Stanford, CA USA; 7 Pacific Health Research and Education Institute, Honolulu, HI USA

**Keywords:** Cognitive reserve, Compensation, Alzheimer’s disease, Dementia, Neuropathologic lesions, Ageing

## Abstract

**Background:**

We have proposed that cognitive resilience (CR) counteracts brain damage from Alzheimer’s disease (AD) or AD-related dementias such that older individuals who harbor neurodegenerative disease burden sufficient to cause dementia remain cognitively normal. However, CR traditionally is considered a binary trait, capturing only the most extreme examples, and is often inconsistently defined.

**Methods:**

This study addressed existing discrepancies and shortcomings of the current CR definition by proposing a framework for defining CR as a continuous variable for each neuropsychological test. The linear equations clarified CR’s relationship to closely related terms, including cognitive function, reserve, compensation, and damage. Primarily, resilience is defined as a function of cognitive performance and damage from neuropathologic damage. As such, the study utilized data from 844 individuals (age = 79 ± 12, 44% female) in the National Alzheimer’s Coordinating Center cohort that met our inclusion criteria of comprehensive lesion rankings for 17 neuropathologic features and complete neuropsychological test results. Machine learning models and GWAS then were used to identify medical and genetic factors that are associated with CR.

**Results:**

CR varied across five cognitive assessments and was greater in female participants, associated with longer survival, and weakly associated with educational attainment or *APOE* ε4 allele. In contrast, damage was strongly associated with *APOE* ε4 allele (*P* value < 0.0001). Major predictors of CR were cardiovascular health and social interactions, as well as the absence of behavioral symptoms.

**Conclusions:**

Our framework explicitly decoupled the effects of CR from neuropathologic damage. Characterizations and genetic association study of these two components suggest that the underlying CR mechanism has minimal overlap with the disease mechanism. Moreover, the identified medical features associated with CR suggest modifiable features to counteract clinical expression of damage and maintain cognitive function in older individuals.

**Supplementary Information:**

The online version contains supplementary material available at 10.1186/s13195-023-01329-z.

## Introduction

We hypothesize that cognitive resilience (CR) counteracts brain damage from neurodegenerative disease(s) such that older individuals who harbor a high burden of disease(s) sufficient to cause dementia remain cognitively normal [1]. CR to Alzheimer’s disease (AD) has been studied most extensively [2], but CR also has been described for prevalent, related diseases that can cause dementia, including Lewy body disease, vascular brain injury, limbic-associated TDP-43 encephalopathy, and hippocampal sclerosis—the so-called AD-related dementias (ADRDs) [3–5]. Traditionally, CR has been defined as an uncommon, categorical trait, i.e., an individual either did or did not meet the criteria for CR [6] (henceforth binary-CR); however, this approach appears to capture only the most extreme examples of CR and neglects its likely variable expression by different people and across different cognitive domains. In addition to these impediments, the overlapping signs and symptoms of AD and ADRDs, as well as limited reliable biomarkers for most ADRDs, confound the accurate assignment of CR [7]. Previously, most of the apparent CR assigned during life derives from lower burden of undetectable comorbid ADRD(s), underscoring the unique value of autopsy-based studies that permit comprehensive assessment of AD and ADRDs and thus assignment of CR [2]. Here we tested the hypotheses that CR may be defined as a continuous trait that varies across cognitive domains, is predicted by modifiable features measurable during life, and is associated with genetic variants.

**Fig. 1:**
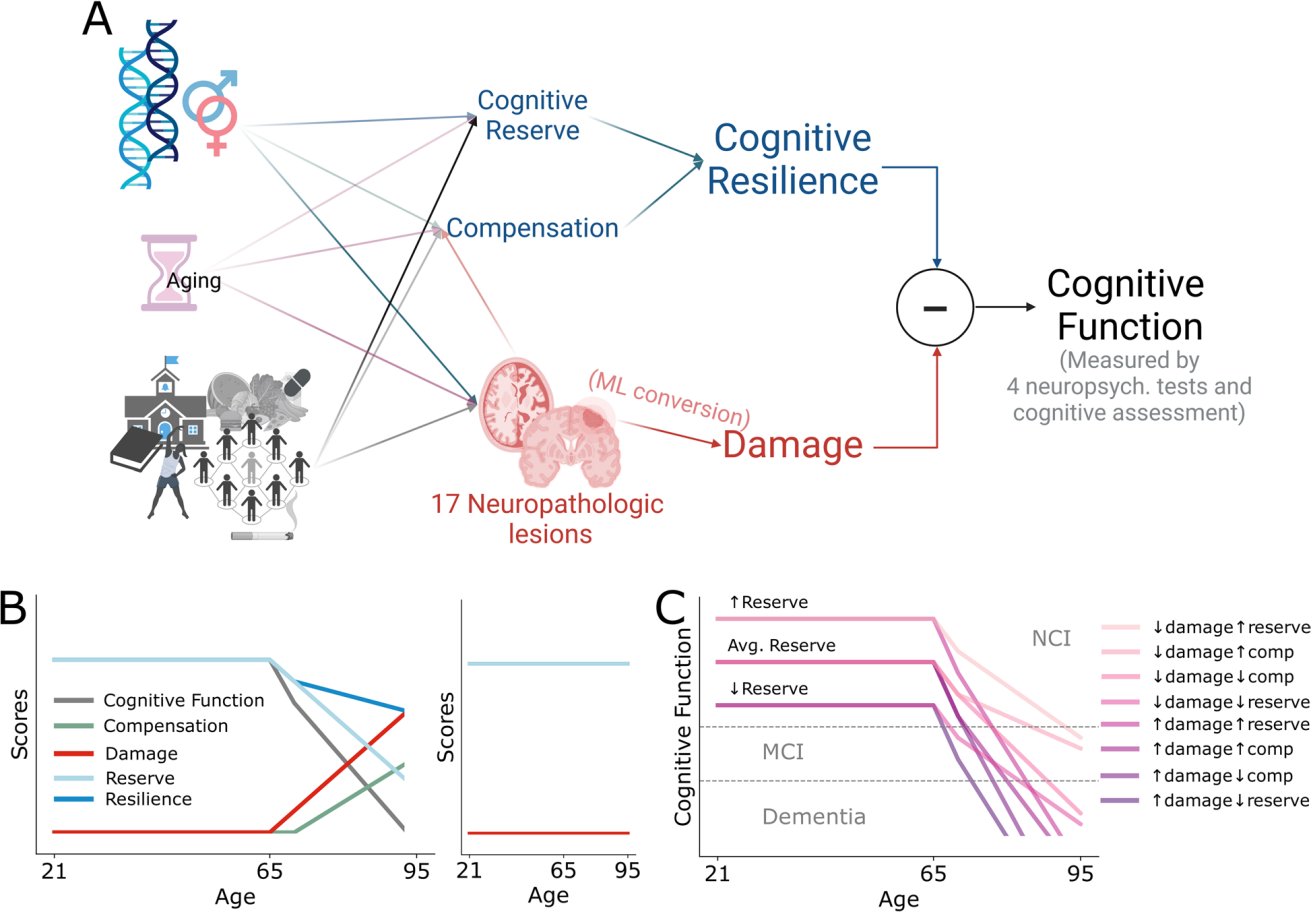
CR score calculation and related terms. **A** CR has two components, reserve and compensation (currently not measured), that along with damage are the result of genetics, aging, and environmental factors. Cognitive function is the outcome of CR minus the impact of brain damage. The strategy for calculating the CR score uses measures of cognitive function from cognitive assessments (four neuropsychological tests and cognitive diagnosis) and the ML-based estimation of damage from consensus ordinal rankings of 17 neuropathologic features of AD and ADRDs. **B** The hypothetical trajectory of CR score and other related terms across the adult lifespan with damage evidenced by pathological changes starting from age of 65. **C** An example trajectory of resistant individuals, i.e., those rare individuals who did not develop damage into late life. **D** Cognitive function trajectories and categorized cognitive status—NCI/MCI/dementia resulting from different scenarios of low vs. high damage, reserve, and compensation

We previously proposed operational definitions and a framework for the relationships among cognitive function, brain damage from AD and ADRDs, and CR with its two subcomponents reserve and compensation [1, 8]. CR can be thought of as the combined impact of cognitive reserve and compensation (Fig. 1A) [8]. Reserve is assembled early in life and is largely unused up to middle age [9, 10]. Later in life, as an individual suffers progressively more damage to the brain from AD and ADRD, cognitive function decreases as does CR, a balance between drawing down reserves and, following a short delay, launching compensatory processes (Fig. 1B left) [1]. Individuals with higher reserve have a greater premorbid capacity to offset damage and are more likely to preserve cognitive function into older ages, an outcome supported by clinical studies showing higher baseline cognitive function is associated with reserve [11] and also with higher cognitive function later in life [12, 13]. The special case where there was no damage is a condition that we have termed resistant (Fig. 1B right) [5, 14]. Based on this, in the current study, we derived simple mathematical relationships to initiate a more rigorous approach to this complex topic with varyingly defined terms and applied them to the expertly annotated NACC dataset. Cognitive function was measured by five cognitive assessments (four neuropsychological tests and cognitive diagnosis), to solve for CR score once brain damage was expressed in the same units as cognitive assessments [9].

## Materials and methods

### Cohort description

Data were taken from the National Alzheimer’s Coordinating Center (NACC) data freeze in March 2021, which included data from all individuals with brain autopsies (6518 individuals total) [15]. All participants (or representatives) provided written informed consent; all protocols and assessments were performed with approval by the appropriate institutional internal review boards. The variable names for neuropathologic lesions (statistics summarized in Supplementary Table 1) come from NACC Neuropathology Form version 10. The NACC data dictionary linking the NACC acronyms to exact definitions and assessment methods is publicly available online [16]. From the initial dataset, a cohort of 844 individuals (375 female, 469 male; 106 with no cognitive impairment or NCI, 78 with mild cognitive impairment or MCI, and 660 with dementia) was selected because they met the following three eligibility criteria: (i) comprehensive lesion rankings for 17 neuropathologic features [15], (ii) complete neuropsychological test results for five cognitive assessments, and (iii) less than 24-month duration between the neuropsychological assessment and brain autopsy (NACCINT < 24). The five cognitive assessments include four neuropsychological tests: the total number of animals named in a set period of time (ANIMALS), the total number of story units recalled from the current test administration (LOGIMEM), trail-making test B (TRAILB), and WAIS-R Digit Symbol (WAIS). The fifth cognitive assessment was cognitive diagnosis (NACCUDSD) as a measure of overall cognition performance. These specific tests were selected to assess multiple cognitive domains while maximizing the number of participants based on test data availability in this version of the NACC dataset.

For this cohort, the average interval between the last research evaluation and death was 9 ± 6 months, a duration that we have shown previously does not significantly impact interval conversion [15]. Summary statistics of the cognitive assessments (neuropsychological test results and cognitive diagnosis) are shown in Supplementary Table 2. An additional 221 demographic and medical features for the same individuals also were collected in the NACC dataset (Supplementary Table 3).

### Equations and derivation of CR score

CR refers to a trait of high-burden neuropathologic changes without change in cognitive status and is usually a binary categorization. To express CR as a continuous variable, each individual $i$’s CR was defined as a continuous score and calculated for each cognitive assessment $a$, since each neuropsychological test and cognitive diagnosis reflect the influence of multiple overlapping cognitive domains.

CR score was defined by the following equation; note that we purposely put cognitive measure on the left side of the equation as it makes it easier to follow the proposed concept:

1 $$\mathrm{cognitive\ measur}{\mathrm{e}}_{i,a}=\mathrm{CR\ scor}{\mathrm{e}}_{i,a }-\mathrm{damag}{\mathrm{e\ estimate}}_{i,a}$$

where all variables in Eq. 1 must have the same units as the cognitive assessments.

To solve for CR score in each individual $i$ for each cognitive assessment $a$, we (i) measured $\mathrm{cognitive\ }{\mathrm{measure}}_{i,a}$ from the five cognitive assessments and (ii) estimated $\mathrm{damag}{\mathrm{e\ estimate}}_{i,a}$ from the 17 neuropathologic features. ${\mathrm{Damage\ estimate}}_{i,a}$ then is a representation of each individual’s extent of brain injury as measured by neuropathologic lesions from AD and ADRDs.

The $\mathrm{damag}{\mathrm{e\ estimate}}_{i,a}$ was calculated by subtracting the hypothetical cognitive estimate as if the individuals had low-to-no lesions (neuropathologic lesion composite index [5] (NP) of 0) from the actual cognitive estimate as shown by:

2 $$\mathrm{damag}{\mathrm{e\ estimate}}_{i,a}=\mathrm{cognitive\ } {\mathrm{estimate}}_{i,a}-\mathrm{cognitive\ } {\mathrm{estimate}}_{i,a}(\mathrm{NP}=0)$$

We used a random forest model to estimate damage by training the model to estimate cognitive performance from the 17 neuropathologic features: $\mathrm{cognitive\ }{\mathrm{estimate}}_{i,a}$. For $\mathrm{cognitive\ estimat}{\mathrm{e}}_{i,a}(\mathrm{NP}=0),$ the same model was used without retraining but now with neuropathologic lesion features of each individual replaced by the average of those with NP = 0 in the cohort while keeping the individual’s sex and age of death the same. The process was performed with 5-fold cross-validation to ensure generalizability. The predictions from the test set in each cross-validation iteration were aggregated for downstream analysis.

Although not a focus of the present study due to data limitation, previously we have defined two components of CR to counteract damage: cognitive reserve and compensation, related by the equation:

3 $$\mathrm{CR\ scor}{\mathrm{e}}_{i,a}={\mathrm{reserve}}_{i,a}+{\mathrm{compensation}}_{i,a}$$

where cognitive reserve is excess capacity developed years or even decades before damage occurs, such as the density of neurons or synapses, and compensation is adaptive mechanisms deployed after damage begins, such as recruiting existing neural circuits to accomplish additional tasks. Combining Eqs. 1 and 3, a special case can be derived where

4 $$\mathrm{CR\ scor}{\mathrm{e}}_{i,a}={\mathrm{reserve}}_{i,a}=\mathrm{cognitive\ measur}{\mathrm{e}}_{i,a}$$

when there is no damage, i.e., no neuropathological lesions and thereby minimal damage, and hence no compensation.

We used a second random forest machine learning (ML) model to predict an individual’s CR score based on 221 demographic and medical features without using brain autopsy data, date of death, or cognitive assessments so as to identify variables important to CR score collected during life (Supplementary Table 3). As before, a random forest model was used with 5-fold cross-validation to ensure generalizability. The predictions from the test set in each cross-validation iteration were aggregated for downstream analysis.

### Genetic association study

Of all NACC participants, 3,220 had genotype and phenotype available and consisted of 12 genotyped freezes across AD Centers (ADC 1–12). Batches were genotyped using standard Illumina protocols and performed by the AD Genetics Consortium (ADGC). ADC samples were genotyped and analyzed in separate batches. ADC1 and ADC2 were genotyped using Illumina 660W-Quad arrays, ADCs 3–8 (grouped as “OMNI”) using the Illumina OmniExpress, and ADCs 9–12 (grouped as “GSA”) using Illumina Global Screening Assay (GSA). The demographic of the cohort is shown in Supplementary Table 4.

The standardized ADGC quality control pipeline was performed on the sample and variant levels. Briefly, samples or variants with low call rates (sample missingness > 2%, variant missingness > 5%), sex discordance, or deviations from Hardy–Weinberg Equilibrium were filtered out. A relatedness check was carried out with PC-AiR to avoid false positives [17]. The analysis revealed some identical pairs (kinship ≥ 0.480) which were subsequently dropped, to avoid the uncertainty of whether it was the same person genotyped twice or a pair of twins. For pairs of first-degree kinship (0.177 ≤ kinship < 0.480), only one individual from each related pair (whichever was in a larger ADC batch) was kept. Principal components analysis was conducted using PC-AiR to account for population substructure [18]. Outliers for genetic ancestry (> 5 standard deviations from the mean) were dropped. The samples were imputed with the Trans-Omics for Precision Medicine (TOPMed) program server [19]. Genetic variants with MAF > 0.01 and imputation quality score *R*^2^ > 0.40 were kept for association analysis.

Tests of association between the five CR scores, one for each of the five cognitive assessments, and genetic variants were conducted in each batch separately (A1, A2, OMNI, GSA) by using linear regression performed using RVtests, a tool for rare variant association analysis using sequence data [20]. Association analyses included the following covariates: sex, age at death, years of education, and the first three PCs. Batch-specific results were then combined across datasets in a fixed-effect meta-analysis with an inverse-variance weighted approach, as implemented in METAL [21]. Genetic variants appearing in less than 25% of samples were excluded from the analysis. QQ plots and genomic inflation factors were generated for each resilience score measure (Supplementary Fig. 1).

Genome-wide significant loci identified from the meta-analysis were subjected to functional annotation and scoring using Functional Mapping and Annotation (FUMA, v1.5.2), GeneHancer, Protein Atlas, combined annotation-dependent depletion (CADD), and RegulomeDB [22–24].

## Results

### Calculating estimated damage and continuous CR scores

Figure 1 displays our approach to defining cognitive resilience and its relationship to reserve, compensation, and cognitive performance. Through these admittedly simple definitions, the traditional binary CR concept is transformed into a CR score, deriving from reserve alone or combined with compensation. Figure 1C illustrates hypothetical trajectories in cognitive function based on these linear relationships for different combinations of damage and CR. Supplementary Figure 2 presents the trajectories of all terms in this plausible mix of cognitive function trajectories over the adult lifespan. To demonstrate this with actual data, we used a random forest ML model to calculate the $\mathrm{damag}{\mathrm{e\ estimate}}_{i,a}$ by Eq. 2. To estimate the damage to the brain, the model was first given the 17 neuropathologic profiles, age, and sex as input to predict the cognitive assessment results as the output ($\mathrm{cognitive\ }{\mathrm{estimate}}_{i,a}$ in Eq. 2). Then, the trained model was used to predict the hypothetical cognitive assessment results of each individual now with their 17 neuropathologic measures altered to be the same as the average from those with NP = 0 (*n* = 62), while maintaining the same sex and age. The latter created an age- and sex-specific baseline for each cognitive assessment by utilizing NP scores from individuals with no brain damage. Subtracting the two gives an individual’s damage estimate expressed in the units of each cognitive assessment (Eq. 2). With known cognitive assessment scores and estimated damage expressed in compatible units, an individual’s CR score was calculated according to Eq. 1.

**Fig. 2:**
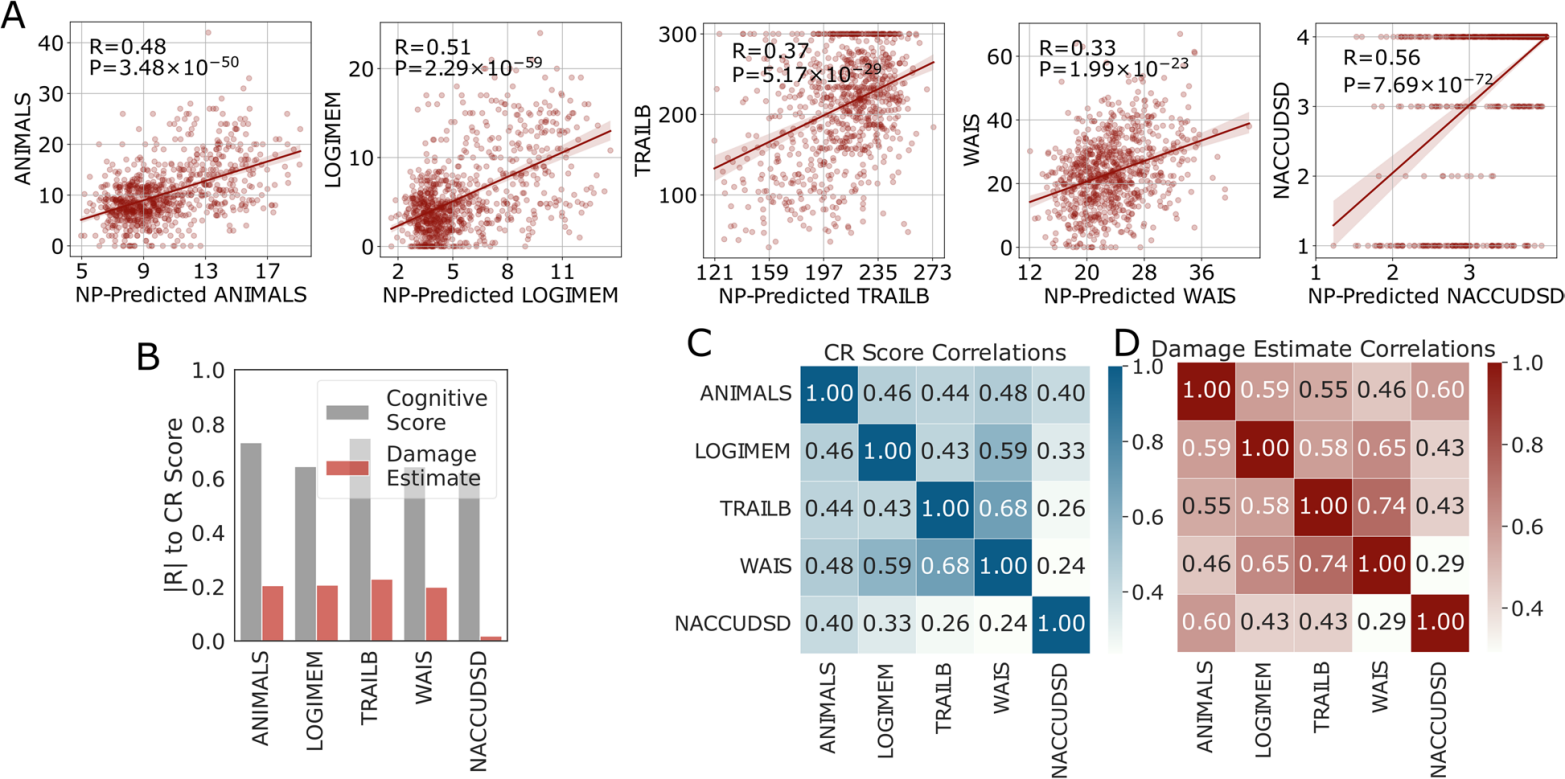
Characteristics of resulting damage and resilience scores. **A** ML prediction performance (Pearson’s *R* and *P* values) of cognitive estimates from Eq. 2 using age, sex, and neuropathologic features. **B** Correlations between CR scores and the corresponding cognitive assessment scores or the damage estimate. **C** Correlation (Pearson’s *R*) of CR scores and **D** damage estimates from different cognitive assessments

The predicted cognitive assessment results from neuropathological features were highly significantly correlated to each of the corresponding actual cognitive assessment results (Fig. 2A). The correlation was lower when using the expanded cohort of 6518 where missing neuropathologic data were imputed with mean values (Supplementary Fig. 3); results from this larger cohort were used only in GWAS where the larger sample size is essential and use of imputed data unavoidable. The distribution of estimated damage from AD and ADRDs is shown for each of the five cognitive assessments in Supplementary Fig. 4A, where larger values indicate more damage.

The distribution of calculated CR scores is shown in Supplementary Fig. 4B. CR scores were correlated with the corresponding neuropsychological test results (Fig. 2B). Generally, higher neuropsychological test scores were correlated with higher CR scores, as expected by Eq. 1. In all neuropsychological tests, participants who were traditionally categorized as resilient (brown dots) tended to have higher CR scores, but they were not completely distinguished from traditionally categorized non-resilient cases (Supplementary Fig. 4C&D). However, by definition, when using the overall cognitive diagnosis, NACCUDSD, all traditionally categorized resilient cases were classified as having normal cognitive (NC). The association between CR scores and damage estimates (Fig. 2B) was much weaker than the association with cognitive assessment results.

### Characteristics of CR scores and their associations

**Fig. 3:**
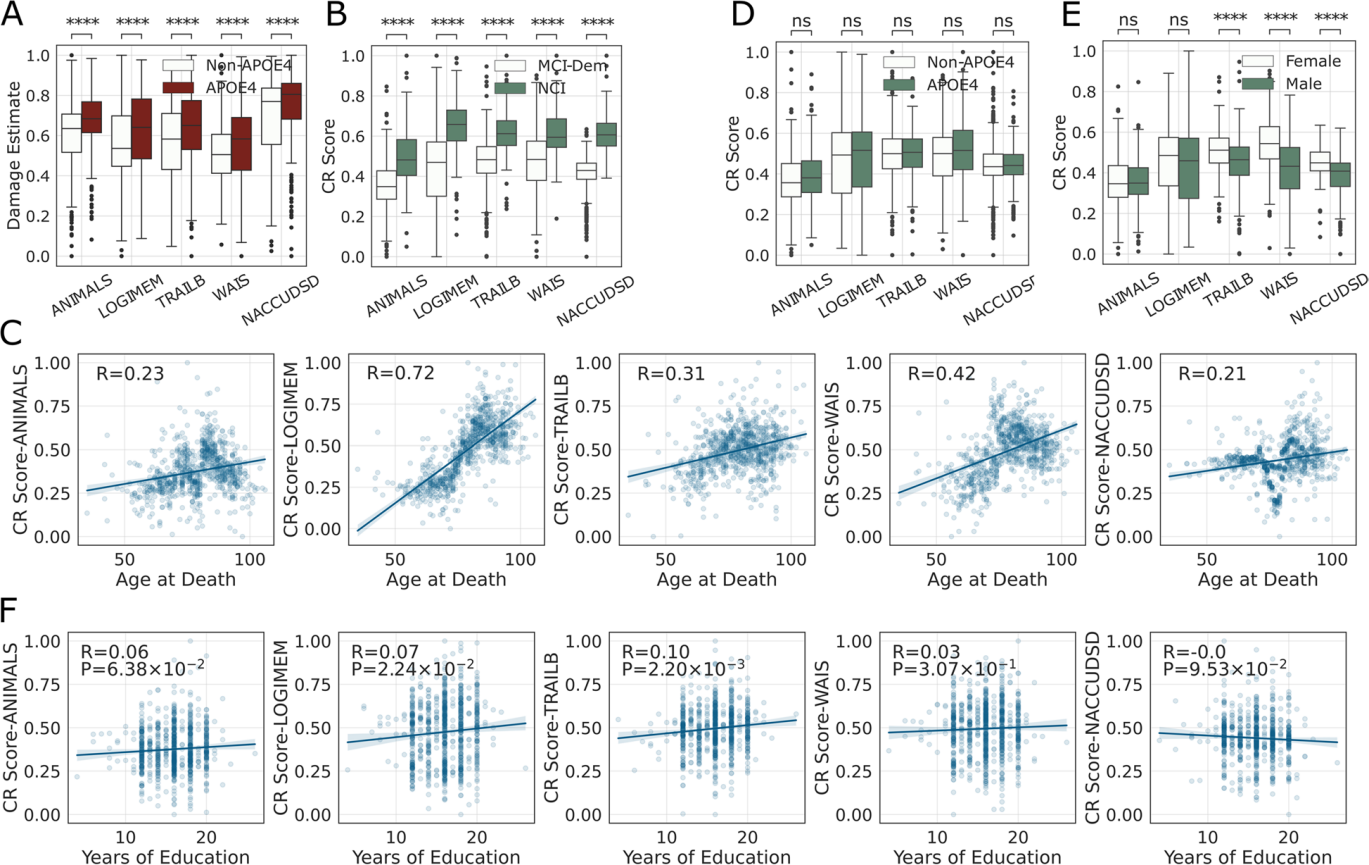
Correlations among CR scores and other features. **A** Stratification of damage estimates by any *APOE* ε4 vs. no *APOE* ε4 (t-test *P* values) for each cognitive assessment (damages were min–max normalized to facilitate visualization). **B** CR scores for each of the five cognitive assessments were stratified by NCI vs. MCI + Dementia groups (t-test *P* values). **C** Correlation (Pearson’s *R* and *P* values) between age at death and CR score as estimated by the five different cognitive assessments. **D** Comparison of CR scores stratified by any *APOE* ε4 vs. no *APOE* ε4 in the main cohort with comprehensive cognitive assessments and neuropathologic features (*n* = 844). **E** CR score stratified by sex (t-test *P* values). **F** Correlation between years of education and CR score as estimated from each of the five cognitive assessments

Correlations among individuals’ CR scores from different cognitive assessments are shown in Fig. 2C. CR scores calculated from TRAILB and WAIS were the two most correlated. CR scores for ANIMALS and LOGIMEM were the two most correlated with the CR score for cognitive diagnosis (NACCUDSD), perhaps because cognitive diagnosis relies heavily on memory performance or varying sensitivity of these measures. The same analyses of damage estimates were largely similar (Fig. 2D). Importantly, the presence of any *APOE* ε4 allele was associated with significantly greater estimated damage for all cognitive assessments (Fig. 3A), with the effect sizes ranging from 0.31 for NACCUDSD to 0.43 for ANIMALS. Damage estimates for *APOE* ε4 homozygotes (*n* = 60) were similar to any *APOE* ε4 allele for all five cognitive assessments but with larger variance because of the limited sample size.

CR scores were higher in individuals diagnosed as cognitively normal (Fig. 3B) and were positively associated with age at death, indicating that individuals who lived longer tended to have higher CR scores (Fig. 3C). In sharp contrast to damage, the effect of *APOE* ε4 on CR scores was not significant for any of the cognitive assessments (Fig. 3D), with effect sizes ranging from − 0.17 for WAIS test and 0.04 for NACCUDSD test. In the expanded cohort of 6518 individuals with mean imputation for the missing neuropathological data, the *APOE* ε4 allele effect was significant for two (LOGIMEM and NACCUDSD) of the five cognitive assessments; however, the effect size of *APOE* ε4 allele on CR score remained small, ranging from 0.00 for WAIS to 0.15 for NACCUDSD (Supplementary Fig. 5). The protective effect of *APOE* ε2 also was focused more on damage than CR (Supplementary Fig. 6).

Stratifying by sex revealed that female participants (who tend to have higher CR [7, 25]) have significantly higher CR scores in TRAILB, WAIS, and NACCUDSD (Fig. 3E). To minimize the possibility of unknown confounders with sex, a linear model was fit using age, sex, education, *APOE* ε4, cognitive status, and severity of AD neuropathologic change (ADNC). The *p*-values of sex in some tests were still strongly significant (Supplementary Table 5), suggesting an actual sex effect.

Years of education were significantly correlated with CR scores as determined for LOGIMEM (*P* = 0.022) and TRAILB (*P* = 0.002, Fig. 3F); however, the apparent effect of educational attainment on CR scores was small with Spearman’s *R* ranging from 0.00 to 0.10 for the five cognitive assessments.

### Demographic and medical feature prediction of CR scores

**Fig. 4:**
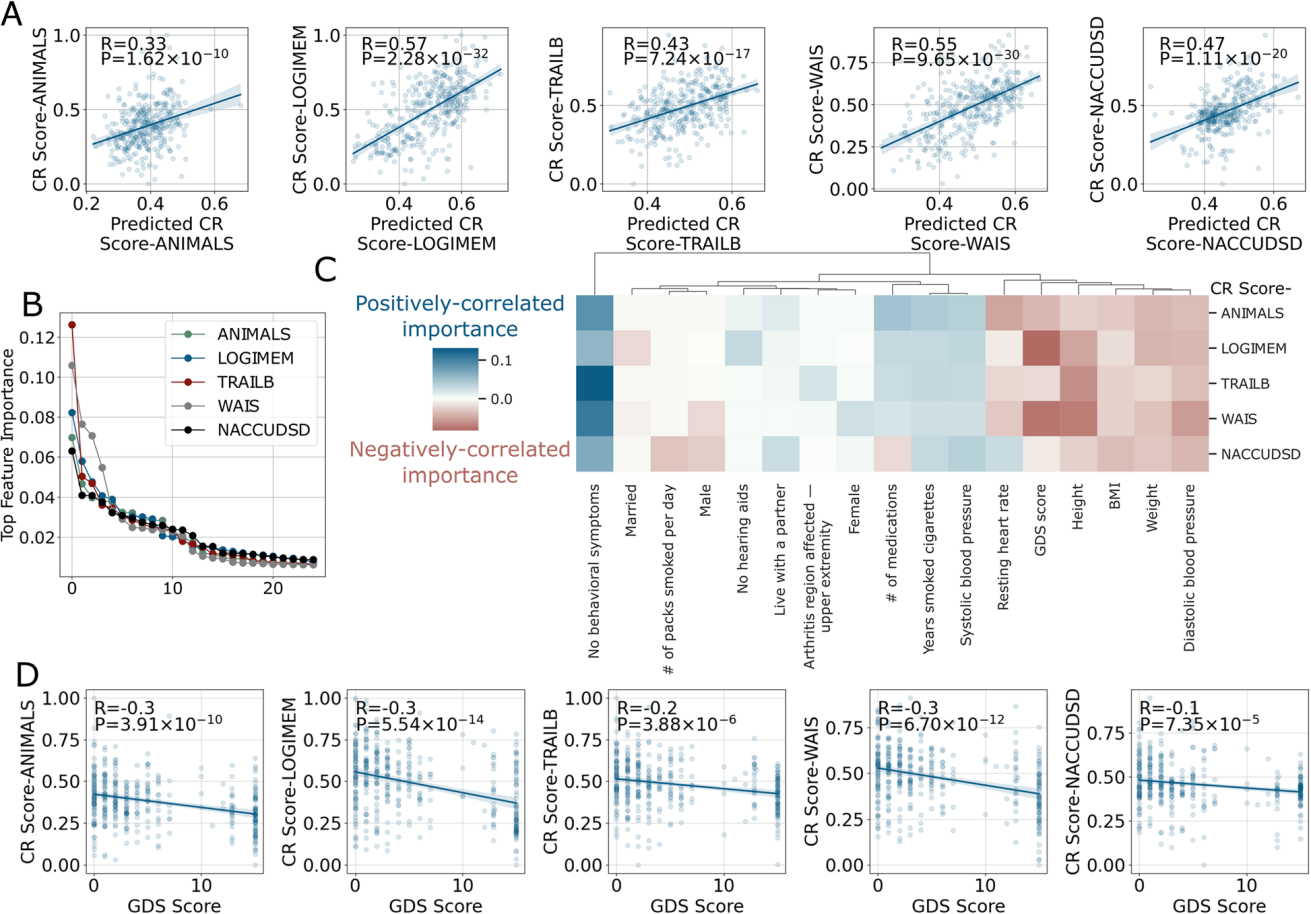
ML prediction of CR values using demographic and medical features and model interpretation results. **A** The performance (Pearson’s *R* and *P* value) of the model in predicting CR values based on data collected during life (not including cognitive diagnosis) scores plotted against CR scores estimated using brain autopsy data. **B** The ranking of feature importance from the random forest model indicated around the top 10 features was the most critical. **C** A heatmap showing the top 10 most important features aggregated from all five cognitive assessments. Negative signs were assigned to feature importance that was negatively correlated with the predicted CR value. **D** Geriatric depression scale (GDS) is shown as an example of an important feature for each of the five cognitive assessments

CR scores were based on neuropathologic data for AD and ADRDs that currently only can be obtained at autopsy. To generalize our work, we next built an ML model to predict CR scores using demographic and medical features obtained during life (excluding cognitive diagnosis and age at death) that were significantly correlated with CR scores (Fig. 4A). The predicted CR scores correlated with the calculated CR scores much more strongly than educational attainment alone. Sorting all items used to predict CR scores by their model feature importance indicated that only about the top 10 features were needed for each of the five cognitive assessments (Fig. 4B); the NACC acronyms of all these features are listed in Supplementary Table 3. Inspecting these top features revealed a pattern of health domains that are important for predicted CR scores (Fig. 4C). The most dominant factor for predicting higher CR scores was related to behavioral symptoms, including lack of depressed mood, psychosis, agitation, or personality change. The severity of depression, as measured by Geriatric Depression Scale (GDS), was correlated with lower predicted CR scores derived from multiple cognitive assessments (Fig. 4D). Potentially related to depression, factors that indicate greater social interactions, such as living with a partner or marital status, also were positively associated with CR scores. Several factors highlighted cardiovascular health as an important indicator of CR score: greater body weight, BMI, heart rate, and diastolic blood pressure were correlated with lower CR scores, and lower systolic blood pressure was correlated with higher CR scores (Fig. 4C). No need for hearing aids or a lower number of medications also were indicators of higher CR scores. Finally, as observed for CR scores, sex was an important feature in predicted CR scores with female participants having greater CR on average than males (Fig. 4C).

Sex-dependent feature importance for CR score was assessed by two ML models, one trained on female-only data and the other on male-only data (see Supplementary Fig. 7 for model performance). The importance for female features is proportional to bubble size, whereas the color is proportional to feature importance for males (Supplementary Fig. 8). Therefore, features that were both strong in color and had bigger sizes were important for both sexes. The sex-dependent features still included a similar set related to cardiovascular health, GDS, and behavioral change as found in the previous all-sex model. However, there were other features that were significant only for females such as Parkinson-related symptoms, and some for males only such as the use of antidepressants or anti-coagulant drugs.

### GWAS associations for some CR scores

GWAS was performed on the expanded cohort of 6518 where missing neuropathologic data were imputed with mean values. This cohort was 46.5% female and comprised individuals with a mean age at death of 82.5 ± 10.2 years and years of education of 15.6 ± 2.9 (Supplementary Table 4). The top signal from the ANIMALS-derived CR score GWAS meta-analysis was in the *MYOM2* <  > LOC101927815 locus (rs182140222, *P* = 6.47 × 10^−11^; Table 1, Supplementary Fig. 9). CR scores of both the LOGIMEM and NACCUDSD identified strong signals in the *APOE* region (rs429358, P-LOGIMEM = 5.84 × 10^−10^, P-NACCUDSD = 1.41 × 10^−12^; Table 1, Supplementary Fig. 10). GWAS of CR score for NACCUDSD also identified two other hits on the LINC02070 <  > *VGLL3* (rs148691207, *P* = 1.33 × 10^−8^; Table 1, Supplementary Fig. 11) and LOC101929692 loci (*P* = 4.44 × 10^−14^). No genome-wide associations were found for the CR score derived from TRAILB or WAIS.

**Table 1:** Genome-wide association (*P* < 5 × 10^−8^) of CR score derived from different cognitive assessments. The 4 symbols per gene in the “Direction” column signify the direction of effect in each cohort in the following order: ADC1, ADC2, OMNI, GSA. The symbol ? indicates that the SNP was not available in the dataset after MAF > 0.01 thresholding,—indicates a negative direction of effect (risk effect of alternate allele), and + indicates a positive direction of effect (protective effect of alternate allele)

CR score	Chr	Position	rs ID	Closest gene	Ref	Alt	Alt Allele Freq	Direction	β (SE)	*P*-value
ANIMALS	8	2201568	rs182140222	*MYOM2* < > LOC101927815	C	G	0.011	??? +	0.096 (0.015)	6.47 × 10^−11^
LOGIMEM	19	44908684	rs429358	*APOE*	T	C	0.274	––	− 0.016 (0.003)	5.84 × 10^−10^
NACCUDSD	3	86714172	rs148691207	LINC02070 < > *VGLL3*	G	A	0.015	+ + + +	0.054 (0.010)	1.38 × 10^−08^
NACCUDSD	6	170624470	NA	LOC101929692	AC	A	0.020	+ ?? +	0.161 (0.021)	4.44 × 10^−14^
NACCUDSD	19	44908684	rs429358	*APOE*	T	C	0.274	––	− 0.017 (0.002)	1.41 × 10^−12^

## Discussion

CR, as defined by our first generation of simple, solvable equations, requires a shift in thinking about AD and ADRDs: the ultimate clinical expression of cognitive impairment is not solely the impact of neurodegeneration, but rather a balance of damage from disease(s) and the counterforce of CR. From this perspective, most clinical, pathologic, and genetic studies of AD and ADRDs have, in fact, investigated the combined influence of damage and CR. Here we attempted to separate these two terms and tested the hypothesis that CR to AD and ADRDs may vary continuously, that CR may differ by cognitive domain, and that this newly defined continuous trait may be predicted by medical features or be associated with genetic variants.

Currently, binary-CR is used when sufficient information on neuropathologic change is present [26, 27]. When post-mortem data is unavailable, some have used normal cognition at old age as a proxy for CR; however, this is confounded by the inclusion of unknown co-morbidities that vary among individuals, and the inclusion of individuals resistant to disease [13]. In other cases, CR has been misused to mean cognitive performance, cognitive reserve, and more, highlighting the importance of clarifying definitions and differences among these related terms [28–30]. Indeed, most of the previous attempts to quantify CR did not reflect a shared definition of CR, but have included individuals’ cognitive scores compared to expected scores at the same pathologic severity [31], indices derived from education and other life factors [25, 32], or image-based deep learning [33, 34] or indices such as brain volume [35]. Importantly, these methods were at best limited to AD only, despite a number of studies suggesting that ADRDs also affect CR [3–5]. In the present study, CR and its relationship to cognitive function and other related terms were clearly defined with different hypothetical scenarios presented. Through this simplified first attempt at mathematical definitions, we calculated CR score based on multiple types of actual cognitive assessment results and extensive knowledge of pathologic changes from AD and ADRDs.

There were several intriguing characteristics of the calculated damage and CR scores. Notably, the level of damage is not the same for each type of cognitive assessment, perhaps reflecting individuals’ shifting alignment between regions of damage and regions that subserve the functions needed for different cognitive assessments. In CR scores, resilient individuals categorized by traditional binary classification did not necessarily have the highest CR scores, underscoring our expectation that a continuous measure of CR may be more informative, especially when comparing across different domains as assessed by various neuropsychological tests. CR scores varied by cognitive assessment, likely reflecting the differing amounts of damage to brain regions and neural circuits that subserve the cognitive functions being tested. As expected, CR scores derived from related cognitive domains tended to be correlated. While all CR scores were positively associated with death at an older age, our results highlighted that the CR score related to the memory domain (LOGIMEM) was the most strongly associated with longer survival (*R* = 0.72). Further, cognitively normal individuals could exhibit higher CR scores, which would indicate higher reserve capacity in our conceptualization. Validating the results of others, CR scores were higher in female participants [6, 36]. Finally, CR scores showed only a weak correlation with damage estimates, suggesting that there may be only limited overlap between mechanisms of CR and mechanisms that drive damage as assessed by these pathologic changes.

Educational attainment has been so widely associated with CR [36–40] that it is sometimes used as a synonymous measure. Our study validated that educational attainment was significantly associated with CR score as measured by some cognitive assessments; however, the effect size was small. Given this modest association between CR score and educational attainment, we sought other demographic or medical features that were strongly predictive of higher CR score in the hope that some might be modifiable. Indeed, feature importance from the random forest model predicting CR scores suggested other life factors might be more important than educational attainment, suggesting that there likely exists superior intra-vitam measures of CR score. These included mental health features such as no behavioral symptoms, no depression, and social interactions, as well as physical health, including no need for hearing aids, cardiovascular health, and the number of medications taken. Although some of these features possibly could represent early symptoms of AD, and thus not be true features of cognitive resilience, they are in agreement with recent studies on the benefits to CR from maintaining good mental health, such as reduced stress at work, conscientiousness [37, 41], and social networks [35, 38], as well as having good cardiovascular health [42]. The impacts of diastolic and systolic blood pressure on our CR scores align with some of the heterogeneous reports of the associations of arterial blood pressure measurements with cognitive decline in older individuals [43]. Although associative and not necessarily deterministic of CR, these data are potentially important because they support lifestyle and medical interventions that may promote CR rather than the more static view of educational attainment, likely a proxy for a complex set of historical events, as contributors to CR.

Several groups have attempted to understand the molecular underpinnings of CR through omics approaches, including genetics [2], epigenetics [44], proteomics [45], and metabolics [29], in order to identify potential interventions or therapeutic targets to mitigate impairment from AD. Focusing on genetics, others have reported a significant association between lower CR and *APOE* ε4, a well-established risk factor for dementia [46, 47]. Our results suggested *APOE* ε4 has a large impact on the calculated damage from multiple neurodegenerative diseases, especially on ANIMAL test. Indeed, previous studies show that semantic fluency in non-demented *APOE* ε4 carriers is already reduced compared to non-carriers [48, 49]. In contrast, we observed a limited impact of *APOE* ε4, or ε2, allele on CR score (no significant association (Fig. 3D) except in the imputed original cohort, which still has minimal effect size (Table 1 and Supplementary Fig. 5). It could be because these studies used different definitions that did not clearly define CR vs. brain damage [27, 50, 51]. In aggregate, our data suggest that APOE ε4’s impact on CR score is much smaller and less pervasive than its impact on damage. This further supports the hypothesis that the mechanisms underlying CR and damage may not overlap substantially.

The validated association of *APOE* ε4 with CR raises the interesting possibility that CR is somehow an indirect measure of less damage from disease(s). Our data do not support this hypothesis. First, from several perspectives, the association underlying damage estimate and CR score were only partially overlapping. Second, the magnitude of the association of *APOE* ε4 with estimated damage was several-fold larger than with CR scores in both the main cohort with comprehensive data and in the expanded cohort with imputed data used for GWAS (Supplementary Table 6). Together, our data support that the apoE4 isoform, a pleiotropic protein with multiple critical functions [52], contributes strongly to damage from AD and ADRDs and makes a significant but much smaller contribution to CR.

Apart from *APOE* alleles, previous investigations into genetic contributions to CR to AD and ADRDs have also associated variants in other genes such as *NLRP3*, *CNOT7*, *MEF2*, and more [27, 50, 51, 53–55]. Even though our cohort was relatively small, and despite the varying definitions of CR across these genetic studies, our results confirmed that inheritance of *APOE* ε4 is associated with lower CR score for LOGIMEM and NACCUDSD by both PCR assay and GWAS array, but not CR score as determined from the other three cognitive assessments. In addition, *MYOM2* was tentatively associated with CR of ANIMALS test; its epigenetic association to AD and neuroinflammatory diseases was reported recently [56, 57]. We also tentatively identified an association between CR with *VGLL3*, which is reported to be associated with autoimmune diseases [58, 59]. Together our results raise the possibility that demographic, behavioral, and medical features may be more strongly determinant of this trait expressed later in life than are genetic variants.

The ML model to estimate damage did not yield a perfect correlation between predicted and actual cognitive assessments (Fig. 2A), which is expected given that outcomes of cognitive assessment are not simply a function of damage but also reflect an individual’s CR (Eq. 1). Therefore, the residuals of the ML model, meaning the difference between the ground truth (actual cognitive assessment results) and the predicted values (predicted cognitive assessments based on damage alone), also could be interpreted as a measure of CR as suggested by previous studies [41, 60]; reassuringly the residuals of the ML model were moderately correlated with CR score (Supplementary Fig. 12). However, the residual method has drawbacks in certain scenarios that need to be considered. For example, a low correlation between neuropathologic features and the cognitive assessment in this method would lead to high correlations (*R* > 0.8) between cognitive assessment results from which the residual was derived [61]. One constraint of our proposed equations is that CR approximates reserve in cases with no damage (Eq. 4). Under such conditions, we observed that an individual’s predicted reserve capacity was highly correlated with actual cognitive assessment results (Spearman’s *R* > 0.8), as expected based on Eq. 4. Importantly, this aligns with others’ hypothesis that premorbid cognitive performance is a determinant of CR [28, 62].

Our study has several limitations. Most importantly is that our equations are simple, a restriction imposed by limited data on the ability to solve for CR. Further, we used linear relationships to approximate resilience, reserve, damage, and function, which may be inaccurate. If we attain means to measure directly CR, reserve, or compensation, then we will be able to solve more complex equations. We selected five measures of cognitive assessment that broadly, but not comprehensively, reflect cognitive function. The accuracy of the calculated CR score hinged on the performance of the ML model to estimate damage from neuropathologic features, restricting us to autopsy-based studies until comprehensive *intra vitam* biomarkers of AD and ADRDs are available. We selected NACC because of its large set of consensus neuropathologic assessments, availability of genomic data, and our previous work with this dataset in predicting neuropathologic lesions [15]. However, this advantage also precluded external validation of our initial model training, due to a lack of similarly expansive, consensus-driven datasets. The measures of damage are based on current consensus neuropathologic guidelines, which have the advantage of providing disease specificity, but may not be the most insightful measures of damage; for example, they lack measures of synaptic injury or rigorous assessment of neuron atrophy or degeneration. Future studies may apply our same approach to other larger cohorts with more sophisticated brain autopsy data, and eventually to longitudinal studies of reliable biomarkers for AD and each ADRD.

The present study outlines a framework to study CR as a continuous variable for multiple cognitive assessments. It also establishes a definitive relationship between CR and other related terms. The study characterized the relationship between damage and CR scores, the balance between which ultimately determines the extent of cognitive impairment. Higher CR scores showed the expected associations to being female and survival to an older age. CR score showed expected associations to *APOE* and educational attainment; however, their effect sizes were modest compared to other potentially modifiable medical and behavioral contributors. Our proposed framework for considering the interaction between damage and CR in determining an individual’s cognitive performance suggests possible challenges to current pharmaceutical approaches and potential opportunities for additional effective interventions.

### Supplementary Information

**Additional file 1: Supplementary Figure 1.** QQ plot for score analysis. **Supplementary Figure 2.** The trajectories of CR score-related components in different theoretical scenarios. **Supplementary Figure 3.** The correlation between predicted neuropsychological test scores and the test scores. **Supplementary Figure 4.** Characteristics of the damage and CR scores. **Supplementary Figure 5.** Comparison of CR scores stratified by any APOE ε4 vs. no APOE ε4 in the main cohort with comprehensive cognitive assessments and neuropathologic features in the expanded cohort with imputed data to be used in GWAS (*n*=6,518). **Supplementary Figure 6.** The protective effect of *APOE* ε2 could come from reducing damage. **Supplementary Figure 7.** The sex-specific model performance and model reduction. **Supplementary Figure 8.** Feature importance from two ML models combined, one trained on female-only data and the other male-only data, to explore sex-specific differences. **Supplementary Figure 9.** The evidence for MYOM2<>LOC101927815 association to CR-ANIMALS. **Supplementary Figure 10.** The evidence for *APOE* association to CR score from LOGIMEM. **Supplementary Figure 11.** The evidence for multiple associations to CR score from NACCUDSD. **Supplementary Figure 12.** The correlation between CR scores and the model’s residual. **Supplementary Table 1.** Cohort description and neuropathologic features. **Supplementary Table 2.** Cohort description with cognitive assessment scores. **Supplementary Table 3.** List of demographic and medical features used for CR score prediction. **Supplementary Table 4.** Demographics and CR scores for each cohort used in the GWAS. **Supplementary Table 5.** Summary statistics from linear regression models associating all primary factors of interest and CR scores from different types of cognitive assessment. **Supplementary Table 6.** Genome-wide association of damage estimates derived from different cognitive assessments.

## Data Availability

The data is available and can be requested from the NACC at https://naccdata.org/requesting-data/data-request-process.
