# Supplementary material for: Quantitative estimate of cognitive resilience and its medical and genetic associations
Source: Alzheimers Res Ther. 2023 Nov 6;15:192. doi: 10.1186/s13195-023-01329-z (PMC10626669; doi:10.1186/s13195-023-01329-z)
Supplement: Supplementary file 1 — Additional file 1: Supplementary Figure 1. QQ plot for score analysis. Supplementary Figure 2. The trajectories of CR score-related components in different theoretical scenarios. Supplementary Figure 3. The correlation between predicted neuropsychological test scores and the test scores. Supplementary Figure 4. Characteristics of the damage and CR scores. Supplementary Figure 5. Comparison of CR scores stratified by any APOE ε4 vs. no APOE ε4 in the main cohort with comprehensive cognitive assessments and neuropathologic features in the expanded cohort with imputed data to be used in GWAS (n=6,518). Supplementary Figure 6. The protective effect of APOE ε2 could come from reducing damage. Supplementary Figure 7. The sex-specific model performance and model reduction. Supplementary Figure 8. Feature importance from two ML models combined, one trained on female-only data and the other male-only data, to explore sex-specific differences. Supplementary Figure 9. The evidence for MYOM2<>LOC101927815 association to CR-ANIMALS. Supplementary Figure 10. The evidence for APOE association to CR score from LOGIMEM. Supplementary Figure 11. The evidence for multiple associations to CR score from NACCUDSD. Supplementary Figure 12. The correlation between CR scores and the model’s residual. Supplementary Table 1. Cohort description and neuropathologic features. Supplementary Table 2. Cohort description with cognitive assessment scores. Supplementary Table 3. List of demographic and medical features used for CR score prediction. Supplementary Table 4. Demographics and CR scores for each cohort used in the GWAS. Supplementary Table 5. Summary statistics from linear regression models associating all primary factors of interest and CR scores from different types of cognitive assessment. Supplementary Table 6. Genome-wide association of damage estimates derived from different cognitive assessments. [file 13195_2023_1329_MOESM1_ESM.docx]

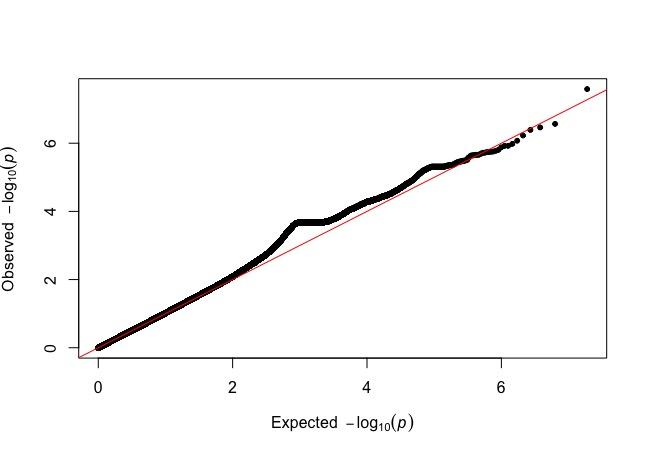

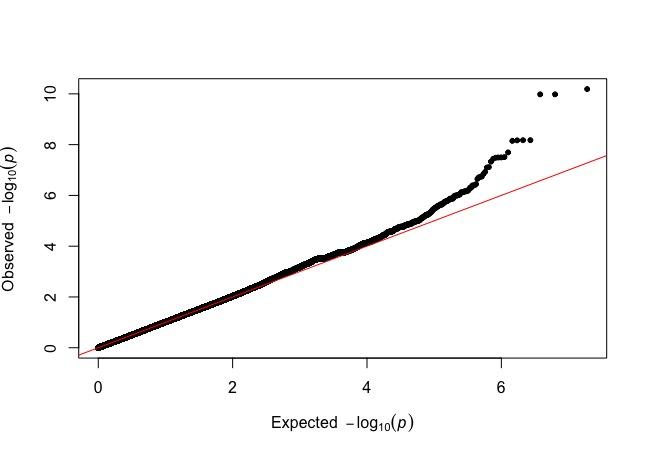

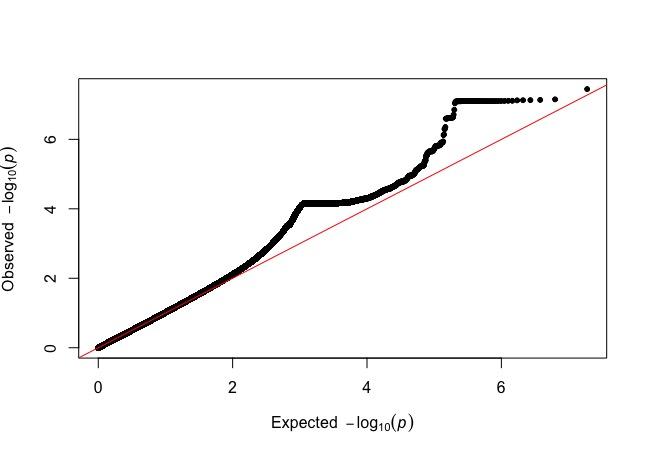

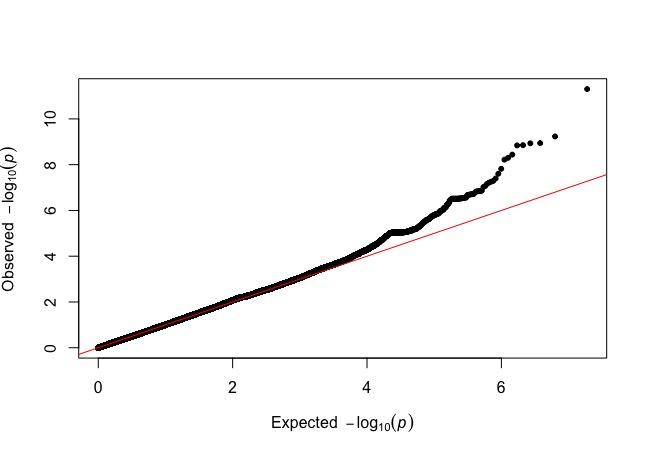

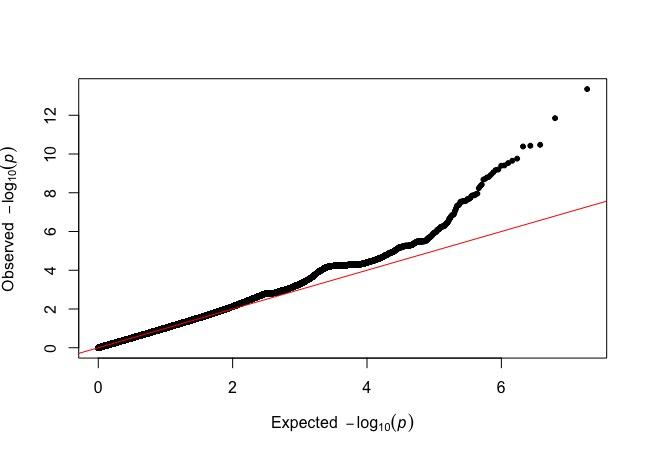


A

B

C

D

E

**Supplementary Figure 1 QQ plot for score analysis.** The plot shows that for all cognitive assessment types, the analysis was not inflated for false positives with GIF of 0.998, 0.992, 0.998, 1.001, and 1.001, for ANIMALS (A), LOGIMEM (B), TRAILB (C), WAIS (D), and NACCUDSD (E).

**Supplementary Figure 2 The trajectories of CR score-related components in different theoretical scenarios.** Trajectories resulting from 8 different hypothetical scenarios. The combination of scenarios was generated from low/high damage with low/high reserve (with average compensation) and with low/high compensation (with average reserve).
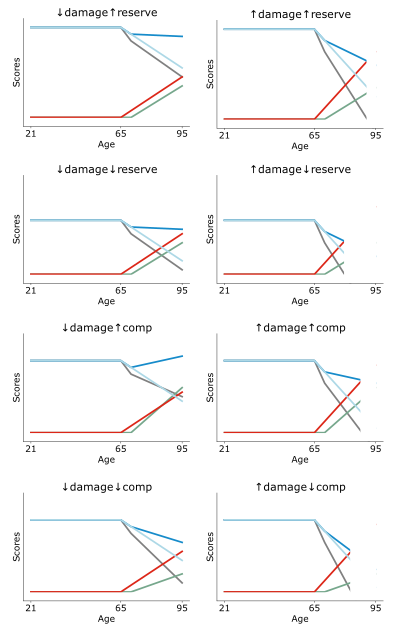


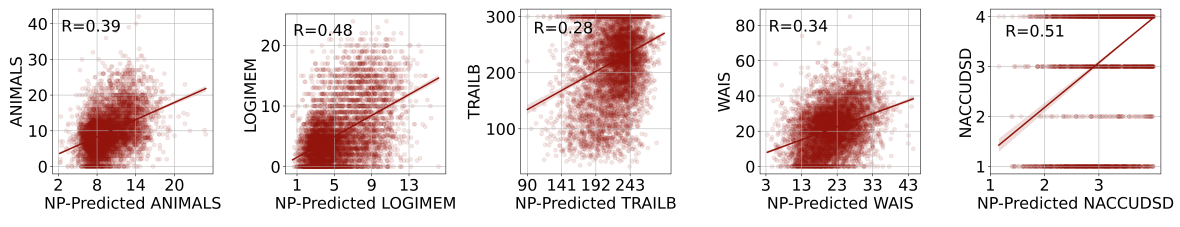


**
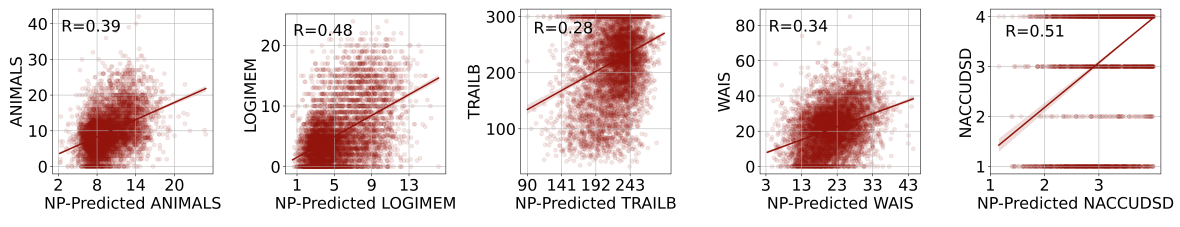
**

**Supplementary Figure 3 The correlation between predicted neuropsychological test scores and the test scores.** The predicted scores from neuropathological lesion information similar to **Figure 2A**, but using the entire cohort of 6,518 instead. In this cohort, missing lesion information was imputed using mean values, and so the predictive power is lower compared to the performance from the selected cohort with complete information shown in **Figure 2A**.


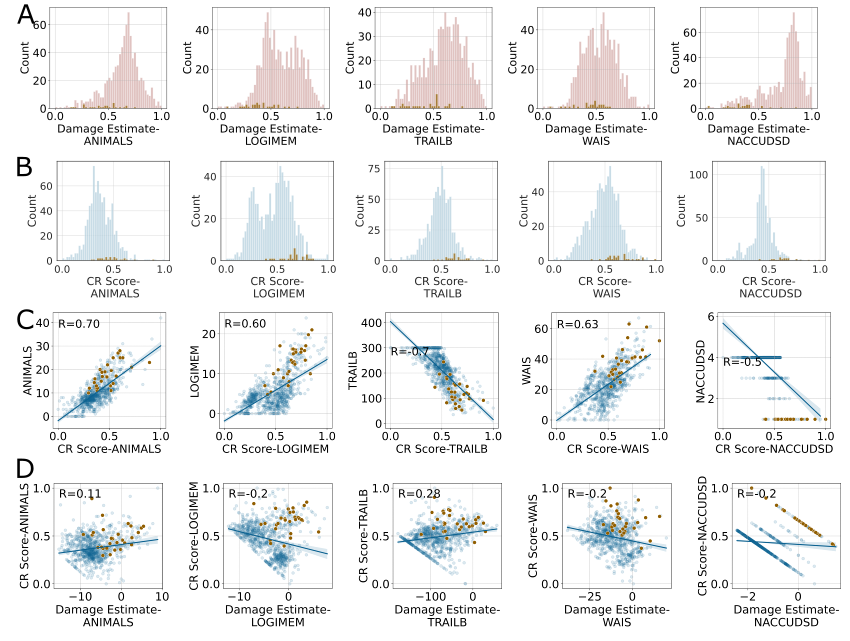


**Supplementary Figure 4 Characteristics of the damage and CR scores.** (**A**) Distributions of $Damag{e Estimate}_{i,a}$ from **Eqn. 2** for each cognitive assessment, where its unit has been transformed to be the same as the cognitive assessment score (higher value means greater the damage). (**B**) Distribution of CR scores. (**C**) Correlations between CR scores and the corresponding cognitive assessment scores. (**D**) Correlations between the CR scores and damage estimate from the corresponding cognitive assessment scores. For (**C**) and (**D**), each dot represents an individual and those colored brown are those who were traditionally categorized as resilient.


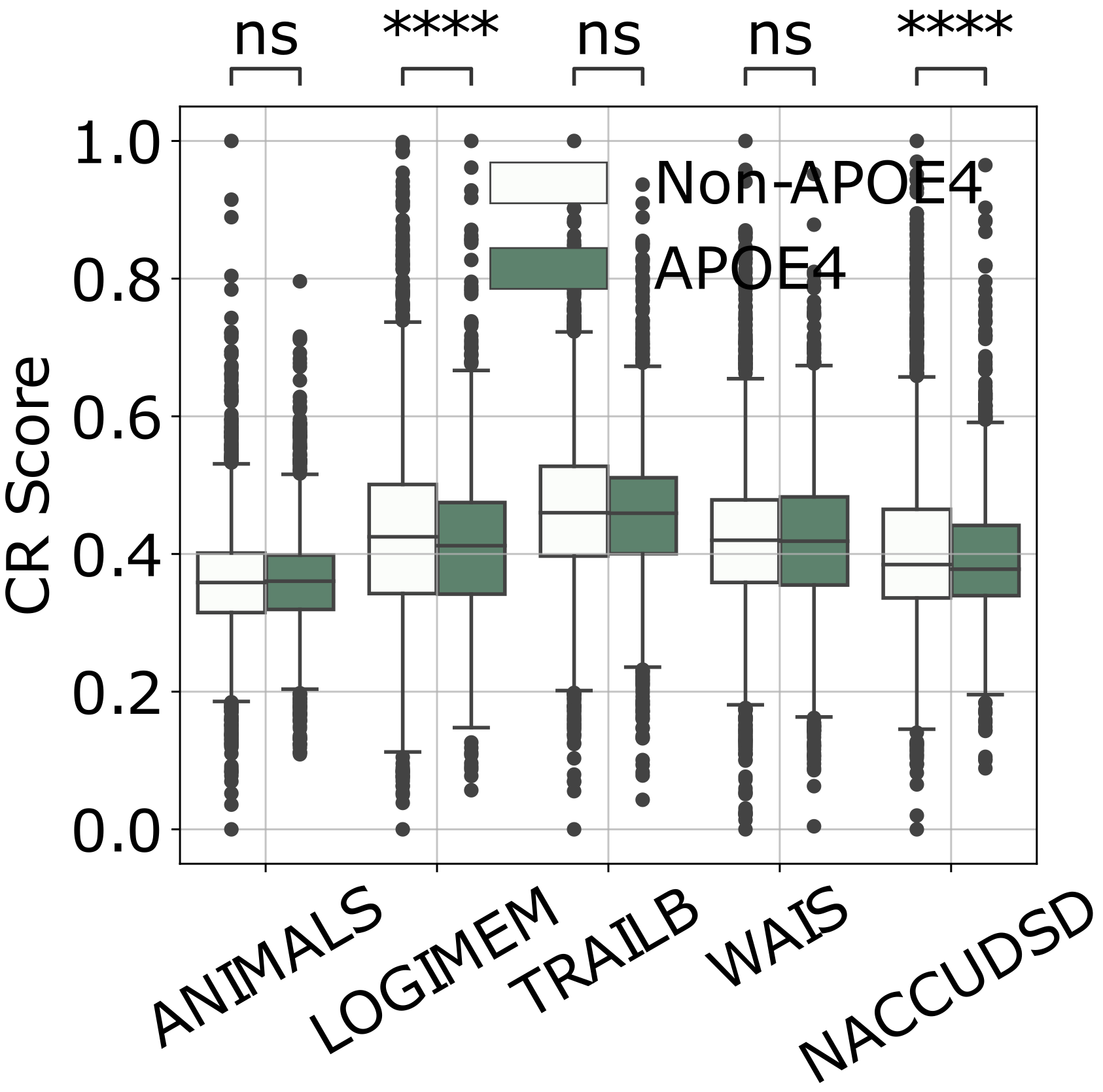


**Supplementary Figure 5** Comparison of CR scores stratified by any APOE ε4 vs. no APOE ε4 in the main cohort with comprehensive cognitive assessments and neuropathologic features in the expanded cohort with imputed data to be used in GWAS (n=6,518).

**
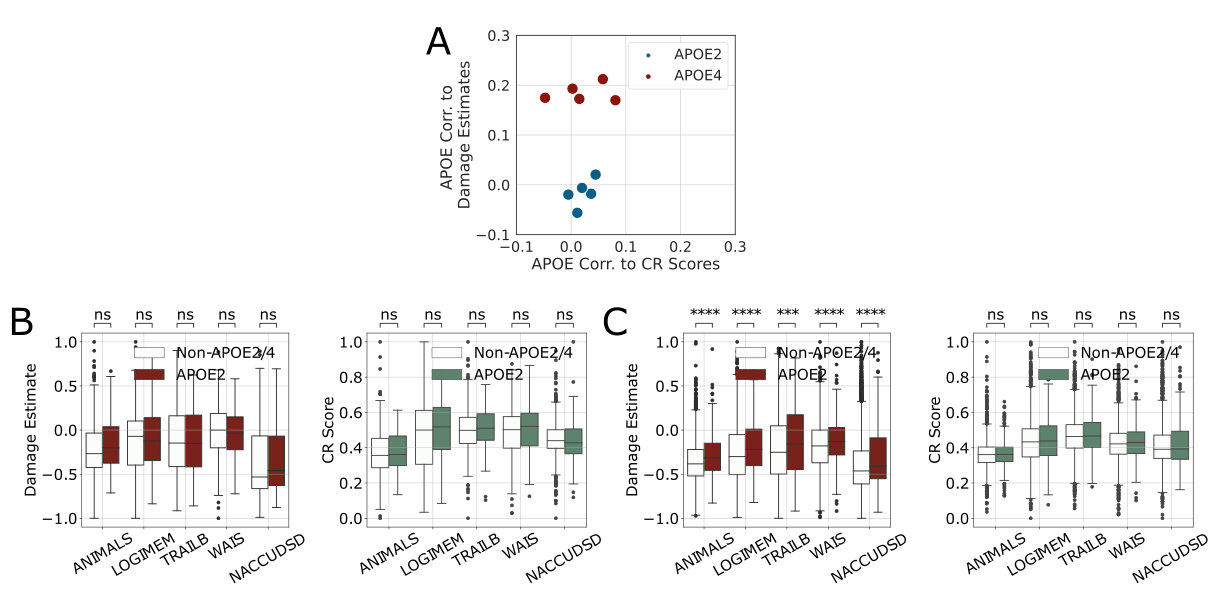
**

**Supplementary Figure 6 The protective effect of *APOE* ε2 could come from reducing damage.** (**A**) The correlation between APOE genotypes with CR score or with estimated damage (APOE4 means any APOE ε4 allele and APOE2 means any APOE ε2 allele with no APOE ε4 allele. Each dot represents a type of cognitive assessment. (**B**) The damage estimate from different cognitive assessments stratified by individuals who had at least one *APOE* ε2 allele versus individuals who were homozygous for the *APOE* ε3 allele in the main cohort of *n=*844. (**C**) The same plot as (**B**) but for the expanded cohort of *n*=8,416 with missing neuropathologic data imputed using mean values.


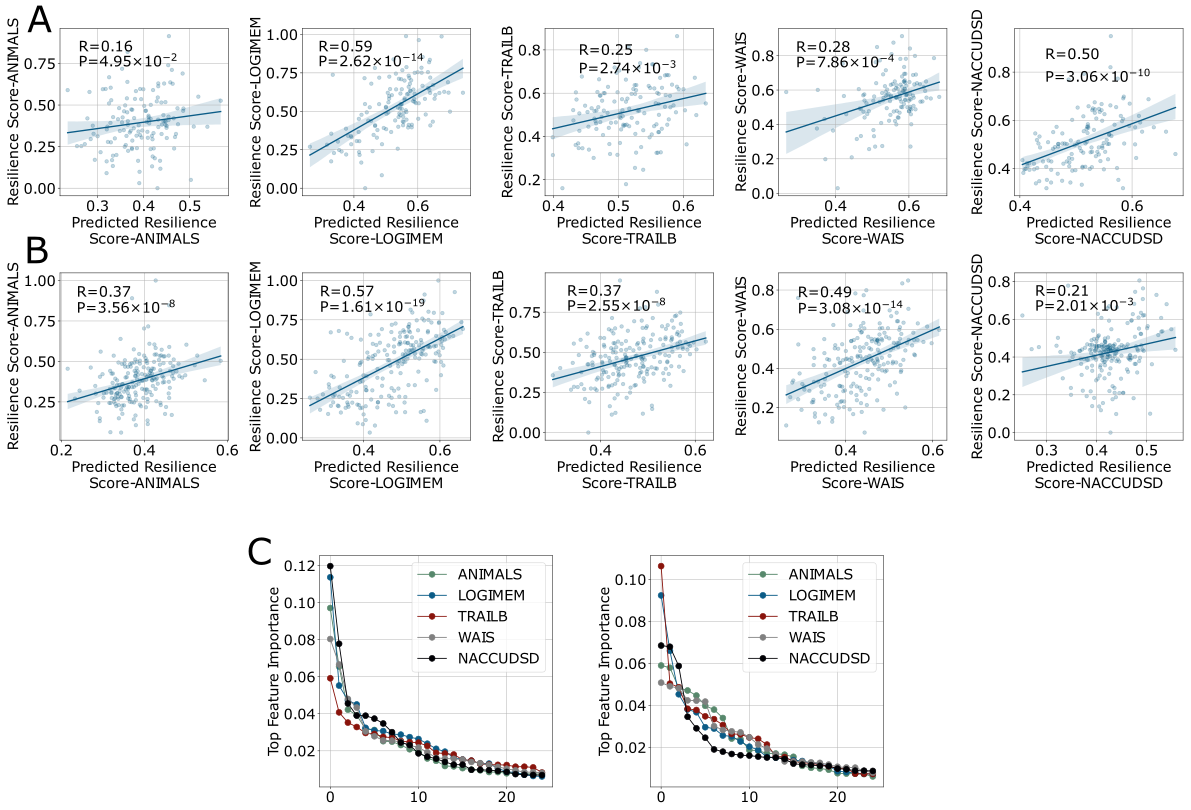


**Supplementary Figure 7 The sex-specific model performance and model reduction.** (**A**) The model performance for predicting CR scores based on demographic and medical features (similar to **Fig. 4**) for females and (**B**) males with (**C**) corresponding model reduction.


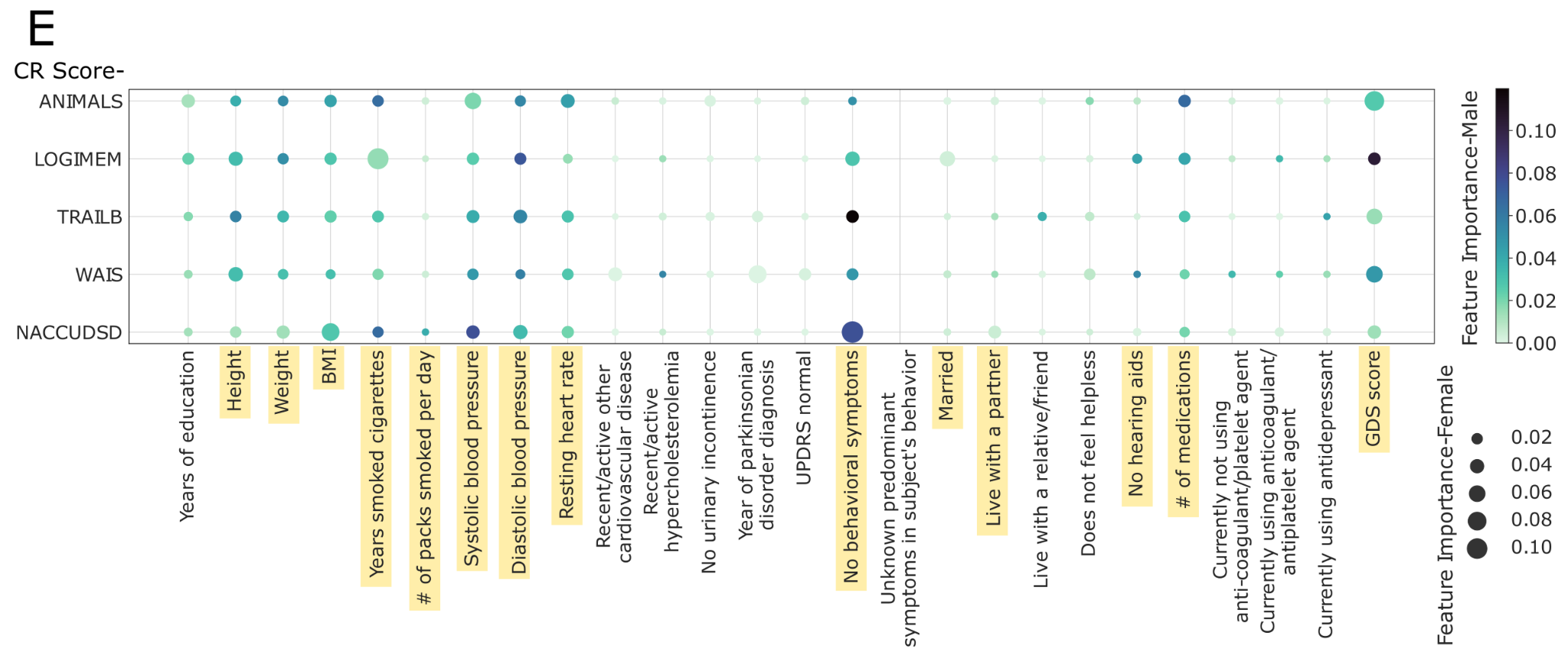
**Supplementary Figure 8** Feature importance from two ML models combined, one trained on female-only data and the other male-only data, to explore sex-specific differences. Features that were important from the all-sex model (**Figure 4C**) were highlighted in yellow.


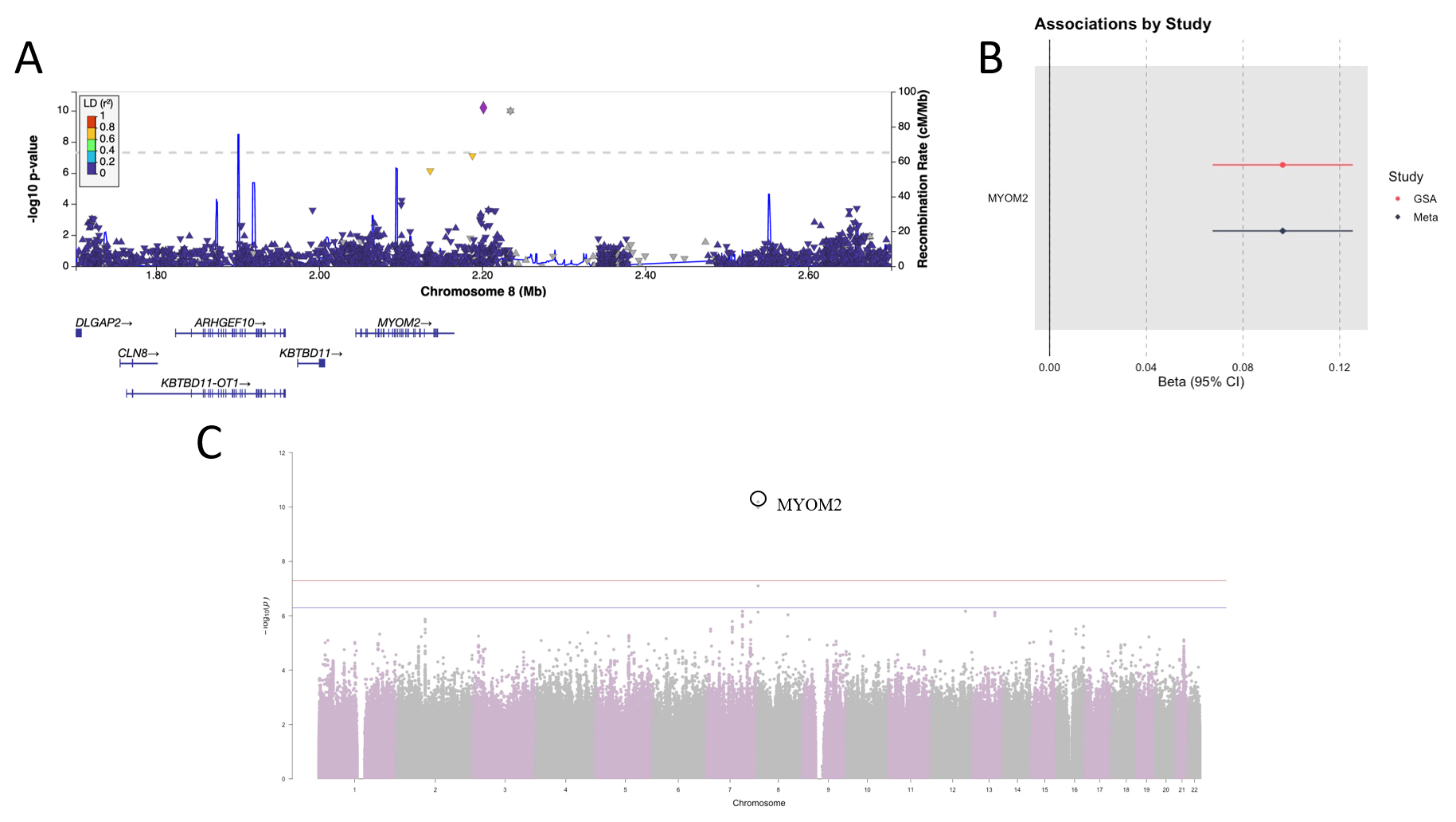

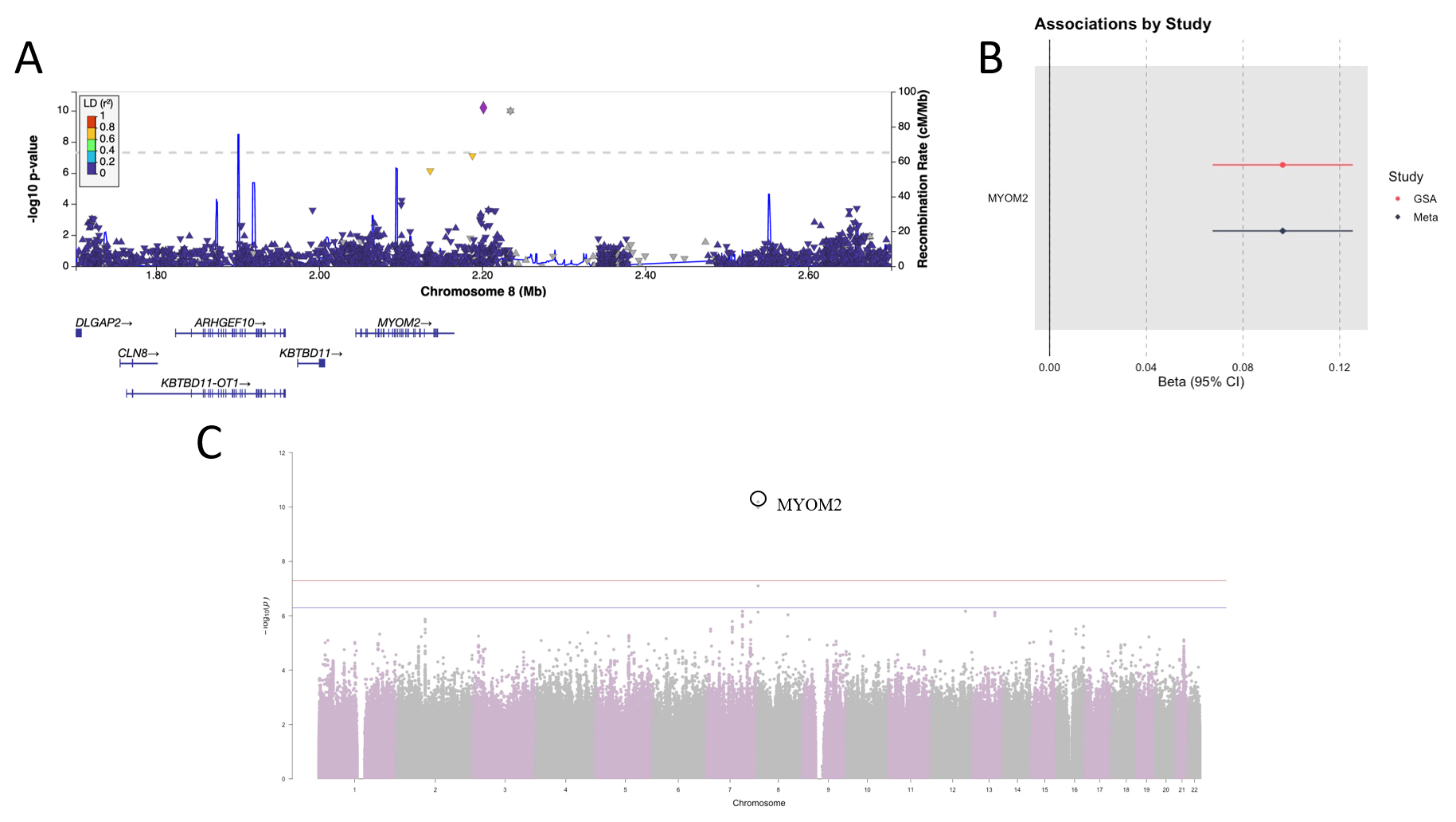

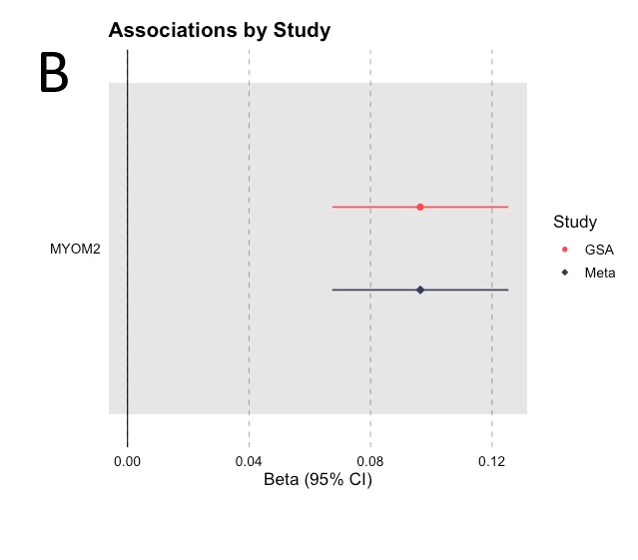


**Supplementary Figure 9 The evidence for MYOM2<>LOC101927815 association to CR-ANIMALS.** (**A**) The regional association plot and (**B**) forest plot for the top loci in the CR score from ANIMALS meta-analysis, and (**C**) the Manhattan plot for the CR score from ANIMALS score analysis.


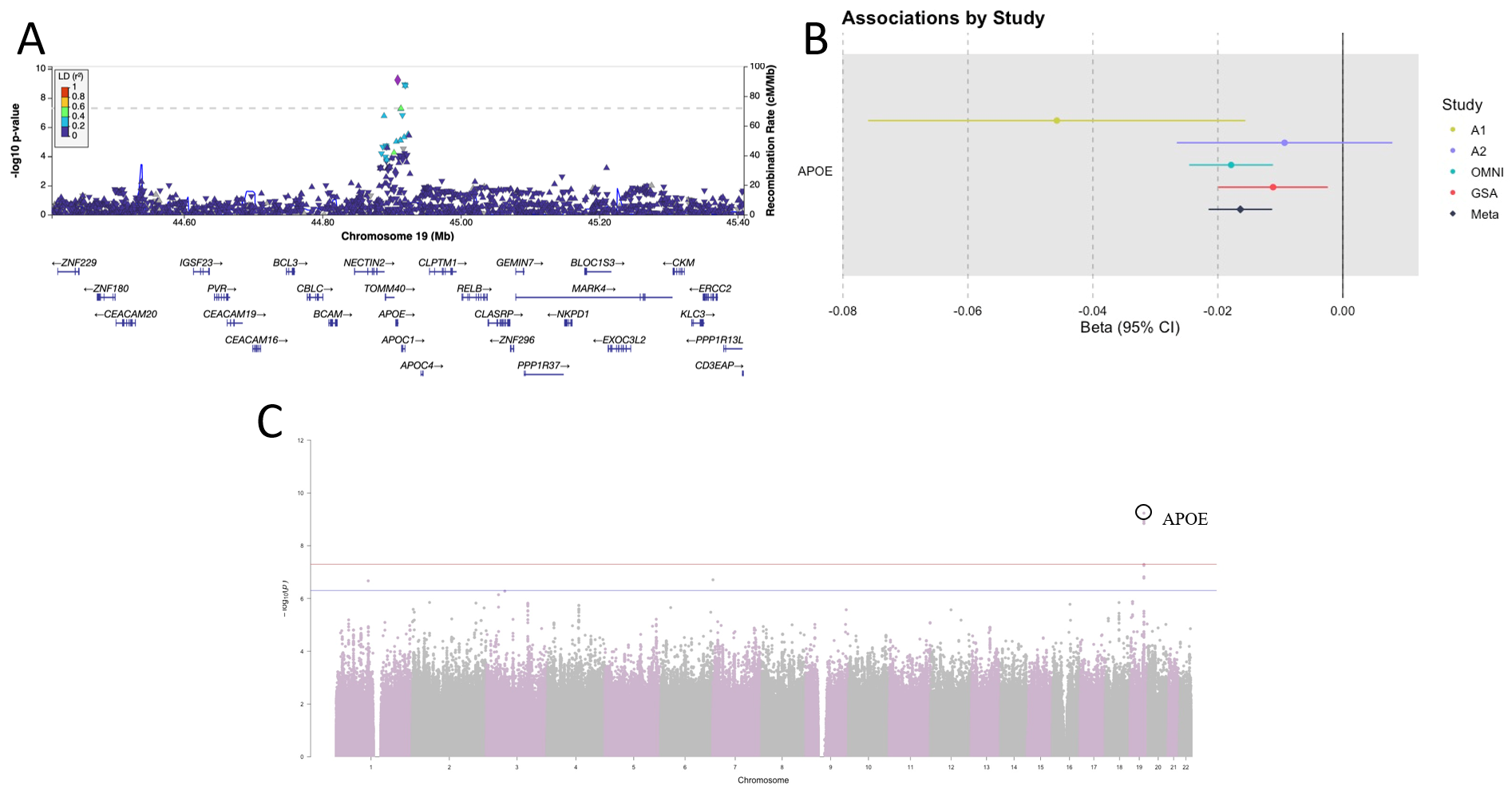

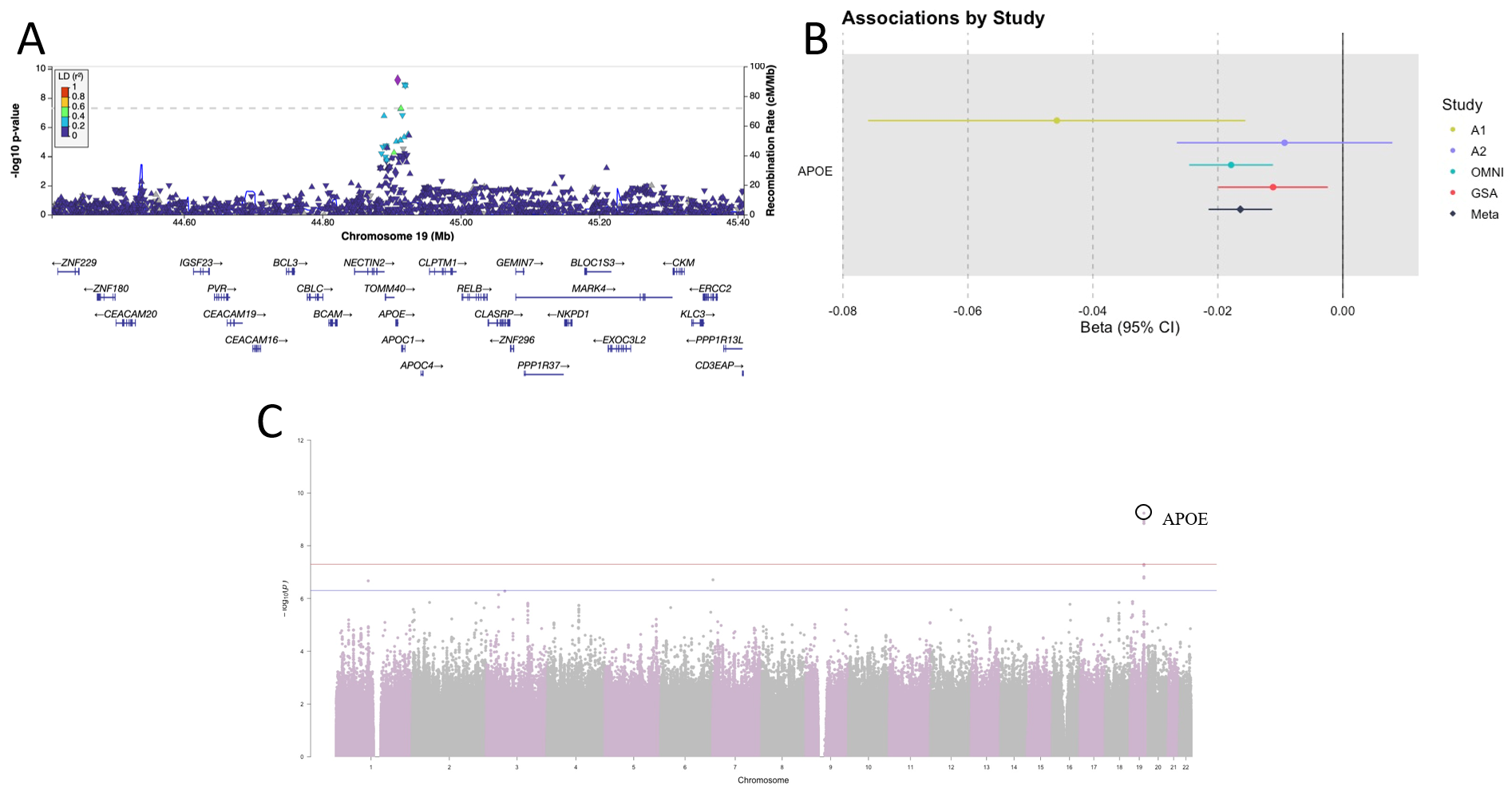

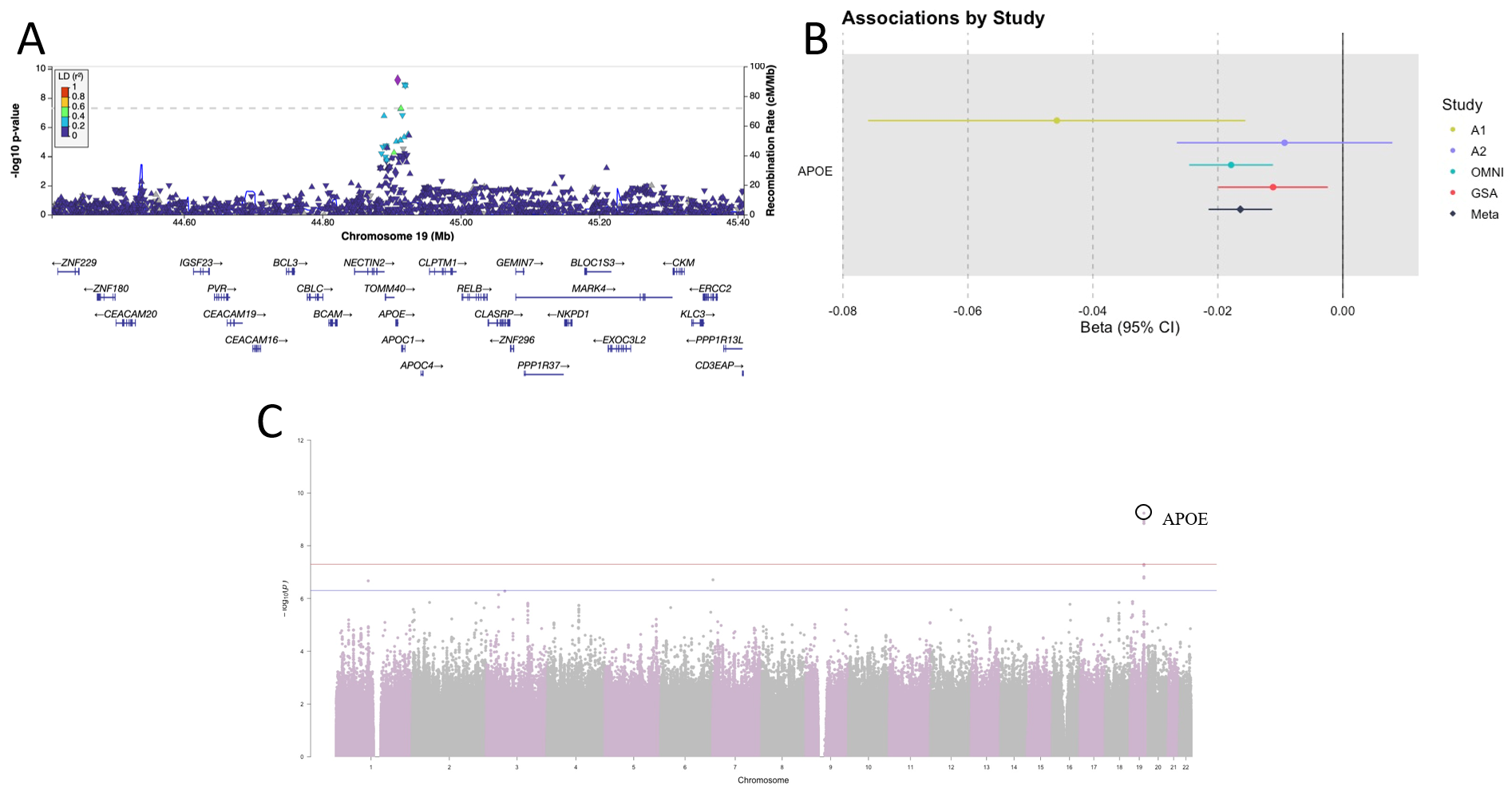


**Supplementary Figure 10 The evidence for *APOE* association to CR score from LOGIMEM.** (**A**) The regional association plot and (**B**) forest plot for the top loci in CR score from LOGIMEM meta-analysis, and (**C**) the Manhattan plot for the CR score from LOGIMEM score analysis.


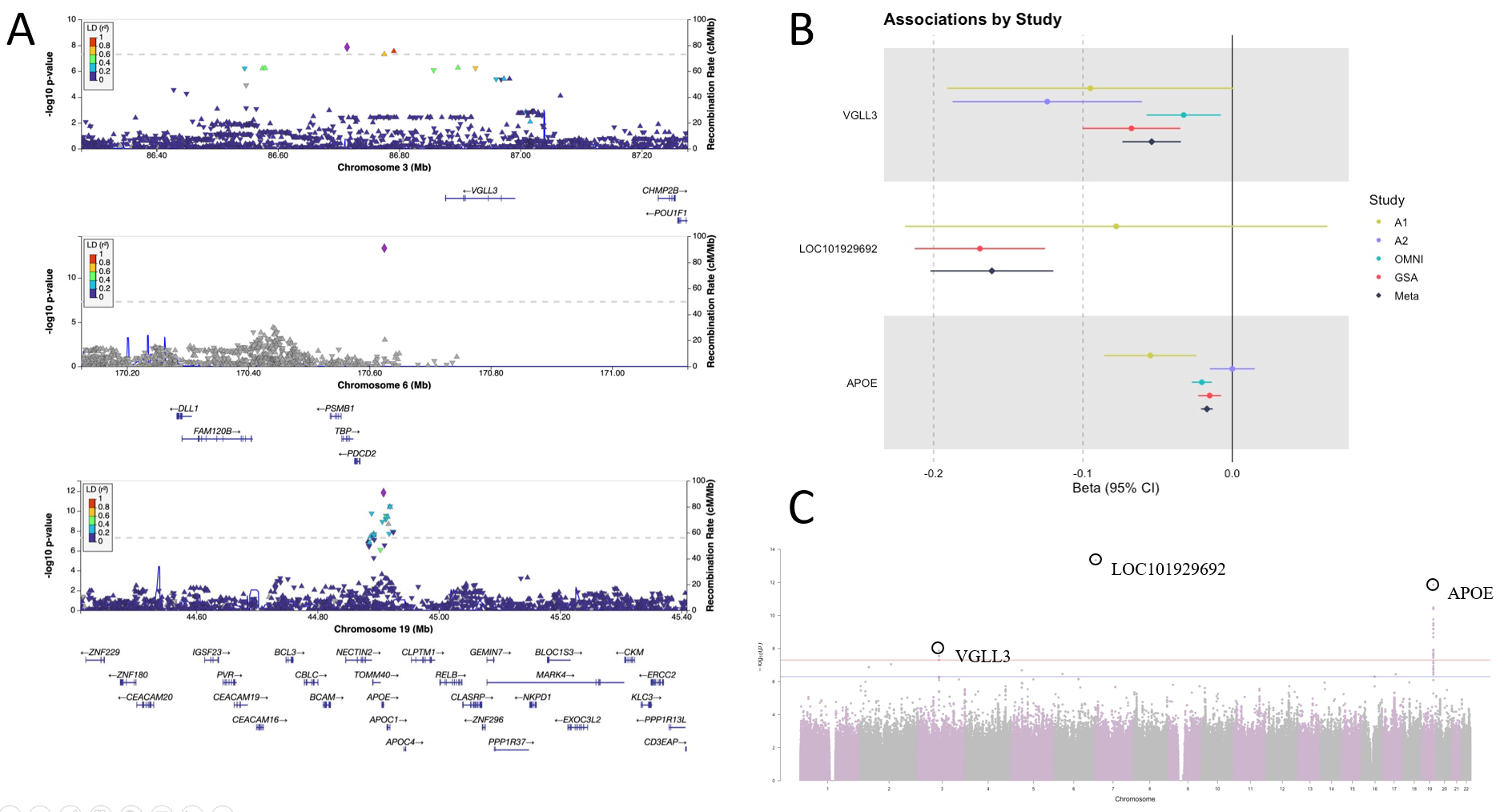

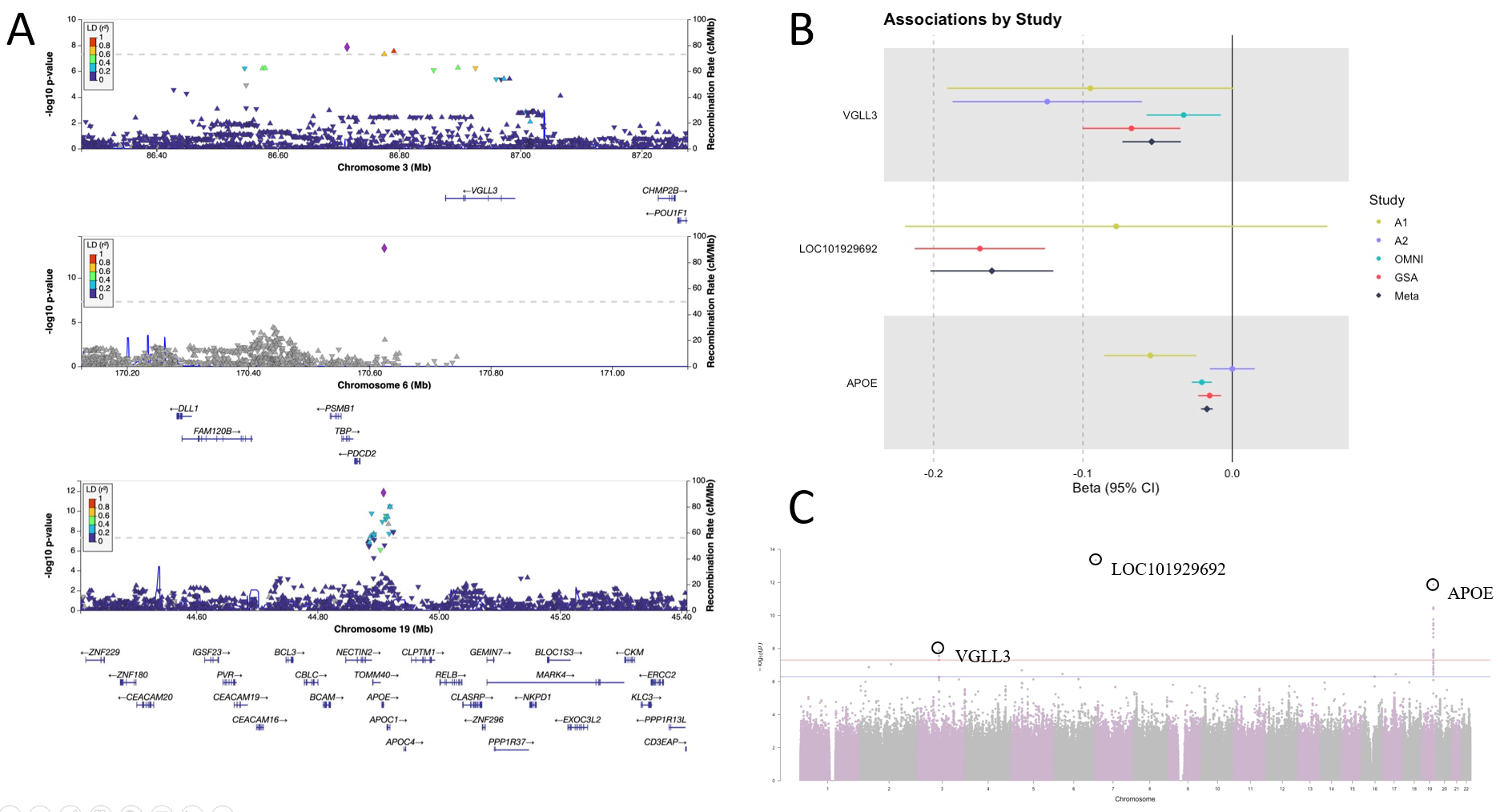


**Supplementary Figure 11 The evidence for multiple associations to CR score from NACCUDSD.** (**A**) The regional association plot and (**B**) forest plot for the top loci in CR score from NACCUDSD meta-analysis, and (**C**) the Manhattan plot for the CR score from NACCUDSD score analysis.


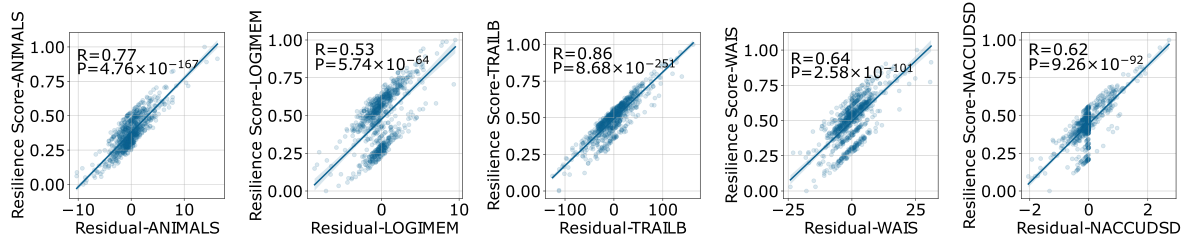


**Supplementary Figure 12 The correlation between CR scores and the model’s residual.** The model’s residual is calculated by the subtraction of ground truth from predicted values, *i.e.* actual cognitive assessment score subtracted from the predicted score based on neuropathologic information.

**Supplementary Table 1** Cohort description and neuropathologic features.

| **NACC VARIABLE** | **DEFINITION** | **LEVEL** | **FEMALE** | | **MALE** | |
| --- | --- | --- | --- | --- | --- | --- |
|  |  |  | **Count** | **%** | **Count** | **%** |
| NACCDAGE | Age at death | Average | 81±12 |  | 77±11 |  |
| ADNC | AD Neuropathologic Change | No | 56 | 15 | 103 | 22 |
|  |  | Yes | 319 | 85 | 366 | 78 |
| NACCBRAA | Braak stage (converted none to VI into 0 to 6) | Average | 4.0±2.0 |  | 3.7±2.1 |  |
| NACCDIFF | Density of diffuse plaques | None | 46 | 12 | 91 | 19 |
|  |  | Sparse | 45 | 12 | 49 | 10 |
|  |  | Moderate | 71 | 19 | 69 | 15 |
|  |  | Frequent | 213 | 57 | 260 | 55 |
| NACCNEUR | Density of neocortical plaques (CERAD score) (C score) | None | 85 | 23 | 149 | 32 |
|  |  | Sparse | 47 | 13 | 53 | 11 |
|  |  | Moderate | 58 | 15 | 63 | 13 |
|  |  | Frequent | 185 | 49 | 204 | 43 |
| NACCAMY | Congophilic amyloid angiopathy | None | 138 | 37 | 193 | 41 |
|  |  | Sparse | 131 | 35 | 139 | 30 |
|  |  | Moderate | 57 | 15 | 76 | 16 |
|  |  | Severe | 49 | 13 | 61 | 13 |
| NACCAVAS | Atherosclerosis | None | 74 | 20 | 116 | 25 |
|  |  | Sparse | 169 | 45 | 210 | 45 |
|  |  | Moderate | 85 | 23 | 95 | 20 |
|  |  | Severe | 47 | 13 | 48 | 10 |
| NACCARTE | Arteriolosclerosis | None | 32 | 9 | 49 | 10 |
|  |  | Sparse | 110 | 29 | 160 | 34 |
|  |  | Moderate | 156 | 42 | 174 | 37 |
|  |  | Severe | 77 | 21 | 86 | 18 |
| NACCHEM | Hemorrhages and microbleeds | No | 348 | 93 | 430 | 92 |
|  |  | Yes | 27 | 7 | 39 | 8 |
| NACCMICR | Microinfarcts | No | 295 | 79 | 375 | 80 |
|  |  | Yes | 80 | 21 | 94 | 20 |
| NPWMR | White matter rarefaction | None | 124 | 33 | 177 | 38 |
|  |  | Sparse | 123 | 33 | 152 | 32 |
|  |  | Moderate | 83 | 22 | 95 | 20 |
|  |  | Severe | 45 | 12 | 45 | 10 |
| NPINF1A | Old infarcts observed grossly — number in cerebral cortex | 0 | 356 | 95 | 444 | 95 |
|  |  | 1 | 14 | 4 | 16 | 3 |
|  |  | 2 | 2 | 1 | 7 | 1 |
|  |  | 3 | 1 | 0 | 0 | 0 |
|  |  | 4 | 0 | 0 | 1 | 0 |
| NPTDPB | pTDP-43 in amygdala | No | 258 | 69 | 330 | 70 |
|  |  | Yes | 117 | 31 | 139 | 30 |
| NPTDPC | pTDP-43 in hippocampus | No | 272 | 73 | 343 | 73 |
|  |  | Yes | 103 | 27 | 126 | 27 |
| NPTDPD | pTDP-43 in entorhinal/inferior temporal cortex | No | 269 | 72 | 345 | 74 |
|  |  | Yes | 106 | 28 | 124 | 26 |
| NPTDPE | pTDP-43 in neocortex | No | 341 | 91 | 411 | 88 |
|  |  | Yes | 34 | 9 | 58 | 12 |
| NACCLEWY | Lewy body disease | None | 245 | 65 | 307 | 65 |
|  |  | Brainstem | 13 | 3 | 9 | 2 |
|  |  | Limbic or amygdala | 74 | 20 | 78 | 17 |
|  |  | Neocortical | 37 | 10 | 64 | 14 |
|  |  | Unspecified | 6 | 2 | 11 | 2 |
| NPHIPSCL | Hippocampal sclerosis (CA1 and/or subiculum) | None | 315 | 84 | 417 | 89 |
|  |  | Unilateral | 8 | 2 | 8 | 2 |
|  |  | Bilateral | 14 | 4 | 13 | 3 |
|  |  | Literality not assessed | 39 | 10 | 31 | 7 |

**Supplementary Table 2** Cohort description with cognitive assessment scores.

| **NACC Variable** | **Definition** | **Female** | **Male** |
| --- | --- | --- | --- |
| **ANIMALS** | Animals — Total number of animals named in 60 seconds | 10.5±5.6 | 10.1±5.7 |
| **LOGIMEM** | Total number of story units recalled  from this current test administration | 5.9±4.6 | 5.4±4.4 |
| **TRAILB** | Trail Making Test Part B — Total  number of seconds to complete | 207±66 | 213±66 |
| **WAIS** | WAIS-R Digit Symbol | 24.3±11.6 | 22.7±11.2 |
| **NACCUDSD** | Cognitive status at UDS visit (NCI/MCI/Dem.) | 49/44/376 | 57/34/284 |

**Supplementary Table 3** List of demographic and medical features used for CR score prediction. Bolded features were among the top ten identified in **Fig. 4**. *Sex is categorized as male or female per the NIH (https://www.nih.gov/nih-style-guide/sex-gender-sexuality).

| **Acronym** | **Short Descriptor** |
| --- | --- |
| **A1 SUBJECT DEMOGRAPHICS** | |
| **SEX** | Subject's sex* |
| HISPANIC | Hispanic/Latino ethnicity |
| HISPOR | Hispanic origins |
| EDUC | Years of education |
| **MARISTAT** | Marital status |
| **NACCLIVS** | Living situation |
| RESIDENC | Type of residence |
| HANDED | Is the subject left- or right-handed? |
| NACCNIHR | Derived NIH race definitions |
| NACCAGE | Subject's age at visit |
| **A3 SUBJECT FAMILY HISTORY** | |
| NACCFAM | Indicator of first-degree family member with cognitive impairment |
| NACCMOM | Indicator of mother with cognitive impairment |
| NACCDAD | Indicator of father with cognitive impairment |
| NACCAM | In this family, is there evidence for an AD mutation (from list of specific mutations)? |
| NACCFM | In this family, is there evidence for an FTLD mutation (from list of specific mutations)? |
| NACCOM | In this family, is there evidence for a mutation other than an AD or FTLD mutation? |
| NACCFADM | In this family, is there evidence of a dominantly inherited AD mutation? |
| NACCFFTD | In this family, is there evidence for an FTLD mutation? |
| **A4 SUBJECT MEDICATIONS** | |
| NACCAAAS | Reported current use of an antiadrenergic agent |
| NACCAANX | Reported current use of an anxiolytic, sedative, or hypnotic agent |
| NACCAC | Reported current use of an anticoagulant or antiplatelet agent |
| NACCACEI | Reported current use of an angiotensin converting enzyme (ACE) inhibitor |
| NACCADEP | Reported current use of an antidepressant |
| NACCAHTN | Reported current use of any type of antihypertensive or blood pressure medication |
| **NACCAMD** | Total number of medications reported at each visit |
| NACCANGI | Reported current use of an angiotensin II inhibitor |
| NACCAPSY | Reported current use of an antipsychotic agent |
| NACCBETA | Reported current use of a beta- adrenergic blocking agent (Beta-Blocker) |
| NACCCCBS | Reported current use of a calcium channel blocking agent |
| NACCDBMD | Reported current use of a diabetes medication |
| NACCDIUR | Reported current use of a diuretic |
| NACCEMD | Reported current use of estrogen hormone therapy |
| NACCEPMD | Reported current use of estrogen + progestin hormone therapy |
| NACCHTNC | Reported current use of an antihypertensive combination therapy |
| NACCLIPL | Reported current use of lipid lowering medication |
| NACCNSD | Reported current use of nonsteroidal anti-inflammatory medication |
| NACCVASD | Reported current use of a vasodilator |
| **A5 SUBJECT HEALTH HISTORY** | |
| TOBAC30 | Smoked cigarettes in last 30 days |
| TOBAC100 | Smoked more than 100 cigarettes in life |
| **SMOKYRS** | Total years smoked cigarettes |
| **PACKSPER** | Average number of packs smoked per day |
| QUITSMOK | If the subject quit smoking, age at which he/she last smoked (i.e., quit) |
| ALCOCCAS | In the past three months, has the subject consumed any alcohol? |
| ALCFREQ | During the past 3 months, how often did the subject have at least one drink of any alcoholic beverage such as wine, beer, malt liquor, or spirits? |
| CVHATT | Heart attack/cardiac arrest |
| HATTMULT | More than one heart attack/cardiac arrest? |
| HATTYEAR | Year of most recent heart attack |
| CVAFIB | Atrial fibrillation |
| CVANGIO | Angioplasty/endarterectomy/stent |
| CVBYPASS | Cardiac bypass procedure |
| CVPACDEF | Pacemaker and/or defibrillator |
| CVPACE | Pacemaker |
| CVCHF | Congestive heart failure |
| CVANGINA | Angina |
| CVHVALVE | Heart valve replacement or repair |
| CVOTHR | Other cardiovascular disease |
| CBSTROKE | Stroke |
| STROKMUL | More than one stroke reported as of the Initial Visit |
| NACCSTYR | Most recently reported year of stroke as of the Initial Visit |
| CBTIA | Transient ischemic attack (TIA) |
| TIAMULT | More than one TIA reported as of the Initial Visit |
| NACCTIYR | Most recently reported year of TIA as of the Initial Visit |
| PD | Parkinson’s disease (PD) |
| PDYR | Year of PD diagnosis |
| PDOTHR | Other parkinsonian disorder |
| PDOTHRYR | Year of parkinsonian disorder diagnosis |
| SEIZURES | Seizures |
| NACCTBI | History of traumatic brain injury (TBI) |
| TBI | Traumatic brain injury (TBI) |
| TBIBRIEF | Traumatic brain injury (TBI) with brief loss of consciousness |
| TRAUMBRF | Brain trauma — brief unconsciousness |
| TBIEXTEN | TBI with extended loss of consciousness — 5 minutes of longer |
| TRAUMEXT | Brain trauma — extended unconsciousness |
| TBIWOLOS | TBI without loss of consciousness — as might result from military detonations or sports injury |
| TRAUMCHR | Brain trauma — chronic deficit |
| TBIYEAR | Year of most recent TBI |
| DIABETES | Diabetes |
| DIABTYPE | If Recent/active or Remote/inactive diabetes, which type? |
| HYPERTEN | Hypertension |
| HYPERCHO | Hypercholesterolemia |
| B12DEF | Vitamin B12 deficiency |
| THYROID | Thyroid disease |
| ARTHRIT | Arthritis |
| ARTHTYPE | Type of arthritis |
| ARTHUPEX | Arthritis, region affected — upper extremity |
| ARTHLOEX | Arthritis, region affected — lower extremity |
| ARTHSPIN | Arthritis, region affected — spine |
| ARTHUNK | Region affected — unknown |
| INCONTU | Incontinence — urinary |
| INCONTF | Incontinence — bowel |
| APNEA | Sleep apnea history reported at Initial Visit |
| RBD | REM sleep behavior disorder (RBD) history reported at Initial Visit |
| INSOMN | Hyposomnia/insomnia history reported at Initial Visit |
| OTHSLEEP | Other sleep disorder history reported at Initial Visit |
| ALCOHOL | Alcohol abuse-clinically significant occurring over a 12-mo period manifested in one of the following areas: work, driving, legal, or social |
| ABUSOTHR | Other abused substances — clinically significant impairment occurring over a 12-month period manifested in one of the following areas: work, driving, legal, or social |
| PTSD | Post-traumatic stress disorder (PTSD) |
| BIPOLAR | Bipolar disorder |
| SCHIZ | Schizophrenia |
| DEP2YRS | Active depression in the last two years |
| DEPOTHR | Depression episodes more than two years ago |
| ANXIETY | Anxiety |
| OCD | Obsessive-compulsive disorder (OCD) |
| NPSYDEV | Developmental neuropsychiatric disorders (e.g., [ASD], attention-deficit hyperactivity disorder [ADHD], dyslexia) |
| PSYCDIS | Other psychiatric disorder |
| **HEIGHT** | Subject’s height (inches) |
| **WEIGHT** | Subject’s weight (lbs) |
| **BPSYS** | Subject blood pressure (sitting), systolic |
| **BPDIAS** | Subject blood pressure (sitting), diastolic |
| **HRATE** | Subject resting heart rate (pulse) |
| **B1 PHYSICAL** | |
| VISION | Without corrective lenses, is the subject's vision functionally normal? |
| VISCORR | Does the subject usually wear corrective lenses? |
| VISWCORR | If the subject usually wears corrective lenses, is the subject's vision functionally normal with corrective lenses? |
| **HEARING** | Without a hearing aid(s), is the subject's hearing functionally normal? |
| HEARAID | Does the subject usually wear a hearing aid(s)? |
| HEARWAID | If the subject usually wears a hearing aid(s), is their hearing functionally normal with a hearing aid(s)? |
| HXHYPER | History or presence of hypertension |
| HXSTROKE | History of stroke |
| NACCBMI | Body mass index (BMI) |
| **B3 UNIFIED PARKINSON'S DISEASE RATING SCALE (UPDRS)** | |
| PDNORMAL | UPDRS normal |
| **B6 GERIATRIC DEPRESSION SCALE (GDS)** | |
| SATIS | Are you basically satisfied with your life? |
| DROPACT | Have you dropped many of your activities and interests? |
| EMPTY | Do you feel that your life is empty? |
| BORED | Do you often get bored? |
| SPIRITS | Are you in good spirits most of the time? |
| AFRAID | Are you afraid that something bad is going to happen to you? |
| HAPPY | Do you feel happy most of the time? |
| HELPLESS | Do you often feel helpless? |
| STAYHOME | Do you prefer to stay at home, rather than going out and doing new things? |
| WONDRFUL | Do you think it is wonderful to be alive now? |
| WRTHLESS | Do you feel pretty worthless the way you are now? |
| ENERGY | Do you feel full of energy? |
| HOPELESS | Do you feel that your situation is hopeless? |
| BETTER | Do you think that most people are better off than you are? |
| **NACCGDS** | Total GDS Score |
| **B8 PHYSICAL/ NEUROLOG EXAM FINDINGS** | |
| PARKSIGN | Parkinsonian signs |
| **B9 CLINICIAN JUDGEMENT OF SYMPTONS** | |
| **NACCBEHF** | Indicate predominant symptom that was first recognized as a decline in the subject’s behavior |
| OTHMUT | Does the subject have a hereditary mutation other than an AD or FTLD mutation? |
| PREVSTK | Previous symptomatic stroke |
| **D1 CLINICIAN** | |
| STROKE | Presumptive etiologic diagnosis of the cognitive disorder — Stroke |
| ESSTREM | Presumptive etiologic diagnosis of the cognitive disorder — Essential tremor |
| DOWNS | Presumptive etiologic diagnosis of the cognitive disorder — Down syndrome |
| HUNT | Presumptive etiologic diagnosis of the cognitive disorder — Huntington's disease |
| PRION | Presumptive etiologic diagnosis of the cognitive disorder — Prion disease (CJD, other) |
| BRNINJ | Presumptive etiologic diagnosis of the cognitive disorder — Traumatic brain injury (TBI) |
| EPILEP | Presumptive etiologic diagnosis of the cognitive disorder — Epilepsy |
| NEOP | Presumptive etiologic diagnosis of the cognitive disorder — CNS neoplasm |
| NEOPSTAT | CNS neoplasm — benign or malignant |
| HIV | Presumptive etiologic diagnosis of the cognitive disorder-Human immunodeficiency virus (HIV) |
| OTHCOG | Presumptive etiologic diagnosis of cognitive disorder- Other neurologic, genetic or infectious condition |
| DEP | Presumptive etiologic diagnosis of the cognitive disorder — Depression |
| DEPTREAT | Depression — Treated or untreated |
| BIPOLDX | Presumptive etiologic diagnosis of the cognitive disorder — Bipolar disorder |
| SCHIZOP | Presumptive etiologic diagnosis of the cognitive disorder — Schizophrenia or other psychosis |
| ANXIET | Presumptive etiologic diagnosis of the cognitive disorder — Anxiety |
| DELIR | Presumptive etiologic diagnosis of the cognitive disorder — Delirium |
| PTSDDX | Presumptive etiologic diagnosis — Posttraumatic stress disorder (PTSD) |
| OTHPSY | Presumptive etiologic diagnosis of the cognitive disorder — Other psychiatric disease |
| ALCABUSE | Current alcohol abuse |
| NACCADMU | Does the subject have a dominantly inherited AD mutation? |
| NACCFTDM | Does the subject have an hereditary FTLD mutation? |
| **D2 CLINICIAN-ASSESSED MEDICAL CONDITIONS** | |
| CANCER | Cancer present in last 12 mths. (excluding non-melanoma skin cancer), primary or metastatic |
| DIABET | Diabetes present at visit |
| MYOINF | Myocardial infarct present within the past 12 months |
| CONGHRT | Congestive heart failure present |
| AFIBRILL | Atrial fibrillation present |
| HYPERT | Hypertension present |
| ANGINA | Angina present |
| HYPCHOL | Hypercholesterolemia present |
| VB12DEF | B12 deficiency present |
| THYDIS | Thyroid disease present |
| ARTH | Arthritis present |
| ARTYPE | Arthritis type |
| **ARTUPEX** | Arthritis region affected — upper extremity |
| ARTLOEX | Arthritis region affected — lower extremity |
| ARTSPIN | Arthritis region affected — spine |
| ARTUNKN | Arthritis region affected — unknown |
| URINEINC | Incontinence present — urinary |
| BOWLINC | Incontinence present — bowel |
| SLEEPAP | Sleep apnea present |
| REMDIS | REM sleep behavior disorder (RBD) present |
| HYPOSOM | Hyposomnia/insomnia present |
| SLEEPOTH | Other sleep disorder present |
| ANGIOCP | Carotid procedure: angioplasty, endarterectomy, or stent within the past 12 months |
| ANGIOPCI | Percutaneous coronary intervention: angioplasty and/or stent within the past 12 months |
| PACEMAKE | Procedure: pacemaker and/or defibrillator within the past 12 months |
| HVALVE | Procedure: heart valve replacement or repair within the past 12 months |
| ANTIENC | Antibody-mediated encephalopathy within the past 12 months |
| **GENETICS** | |
| NACCNURP | Permanently moved to a nursing home |
| NACCAPOE | *APOE* genotype |
| NACCNE4S | Number of *APOE* ε4 alleles |

| **DRUGS** | |
| --- | --- |
| ACETAMINOPHEN | FOLIC ACID |
| ACETAMINOPHEN-HYDROCODONE | GABAPENTIN |
| ASCORBIC ACID | GLUCOSAMINE |
| ASPIRIN | IBUPROFEN |
| CALCIUM ACETATE | MELATONIN |
| CALCIUM CARBONATE | METFORMIN |
| CALCIUM-VITAMIN D | MULTIVITAMIN |
| CONJUGATED ESTROGENS | MULTIVITAMIN WITH MINERALS |
| ESTRADIOL | NIACIN |
| FAMOTIDINE | OMEGA-3 POLYUNSATURATED FATTY ACIDS |
| FERROUS SULFATE | POLYETHYLENE GLYCOL 3350 |
| FINASTERIDE | POTASSIUM CHLORIDE |
| FLUOXETINE | TRAMADOL |
| FLUTICASONE NASAL | UBIQUINONE |
| FLUTICASONE-SALMETEROL | VITAMIN E |

**Supplementary Table 4** Demographics and CR scores for each cohort used in the GWAS.

|  | **A1** | **A2** | **OMNI** | **GSA** | **Meta** |
| --- | --- | --- | --- | --- | --- |
| **Sample size, N (% of meta data)** | 175 (5.4) | 292 (9.1) | 1,796 (55.8) | 957 (29.7) | 3,220 (100) |
| **Age at death, N (%)** | 84.8 (8.8) | 85.1 (7.1) | 83.8 (9.8) | 78.7 (11.1) | 82.5 (10.2) |
| **Female sex, N (%)** | 89 (50.9) | 140 (47.9) | 857 (47.7) | 413 (43.2) | 1,499 (46.5) |
| **Years of education, mean (SD)** | 15.5 (3.4) | 15.3 (2.9) | 15.6 (2.9) | 15.6 (2.8) | 15.6 (2.9) |
| **CR-ANIMALS, mean (SD)** | 0.39 (0.09) | 0.37 (0.08) | 0.37 (0.07) | 0.36 (0.07) | 0.37 (0.07) |
| **CR-TRAILB, mean (SD)** | 0.50 (0.11) | 0.47 (0.10) | 0.48 (0.10) | 0.46 (0.10) | 0.47 (0.10) |
| **CR-WAIS, mean (SD)** | 0.44 (0.12) | 0.43 (0.11) | 0.44 (0.10) | 0.42 (0.10) | 0.43 (0.10) |
| **CR-LOGIMEM, mean (SD)** | 0.48 (0.13) | 0.43 (0.11) | 0.45 (0.11) | 0.42 (0.12) | 0.44 (0.11) |
| **CR-NACCUDSD, mean (SD)** | 0.46 (0.12) | 0.41 (0.09) | 0.43 (0.10) | 0.40 (0.09) | 0.42 (0.10) |

**Supplementary Table 5** Summary statistics from linear regression models associating all primary factors of interest and CR scores from different types of cognitive assessment.

|  |  | CR Score Derived From | | | | |
| --- | --- | --- | --- | --- | --- | --- |
|  |  | ANIMALS | LOGIMEM | TRAILB | WAIS | NACCUDSD |
|  | Adj-R^2^ | 0.91 | 0.95 | 0.95 | 0.95 | 0.95 |
| Age | Coef. | 0.0034 | 0.0089 | 0.0037 | 0.0041 | 0.0032 |
|  | Std. Err. | 0.000 | 0.000 | 0.000 | 0.000 | 0.000 |
|  | P value | 1.67×10^-28^ | 4.94×10^-144^ | 1.01×10^-36^ | 5.03×10^-40^ | 6.72×10^-35^ |
| Sex | Coef. | 0.0220 | -0.0123 | 0.0636 | 0.1091 | 0.1056 |
|  | Std. Err. | 0.008 | 0.008 | 0.008 | 0.008 | 0.007 |
|  | P value | 7.49×10^-03^ | 1.13×10^-01^ | 5.55×10^-16^ | 1.30×10^-37^ | 3.43×10^-47^ |
| Education | Coef. | 0.0093 | -0.0017 | 0.0116 | 0.0062 | 0.0105 |
|  | Std. Err. | 0.001 | 0.001 | 0.001 | 0.001 | 0.001 |
|  | P value | 3.57×10^-13^ | 1.59×10^-01^ | 1.91×10^-21^ | 6.46×10^-07^ | 2.69×10^-22^ |
| *APOE* ε4 | Coef. | 0.0007 | -0.0010 | 0.0038 | 0.0007 | 0.0005 |
|  | Std. Err. | 0.002 | 0.001 | 0.001 | 0.002 | 0.001 |
|  | P value | 6.37×10^-01^ | 5.10×10^-01^ | 8.52×10^-03^ | 6.58×10^-01^ | 7.05×10^-01^ |
| NACCUDSD | Coef. | -0.0288 | -0.0574 | -0.0291 | -0.0343 | -0.0425 |
|  | Std. Err. | 0.004 | 0.003 | 0.003 | 0.004 | 0.003 |
|  | P value | 1.25×10^-14^ | 5.96×10^-53^ | 1.40×10^-16^ | 3.14×10^-20^ | 2.78×10^-39^ |
| ADNC | Coef. | 0.0303 | 0.0309 | 0.0272 | 0.0418 | 0.0140 |
|  | Std. Err. | 0.011 | 0.010 | 0.010 | 0.011 | 0.009 |
|  | P value | 5.08×10^-03^ | 2.70×10^-03^ | 7.42×10^-03^ | 9.69×10^-05^ | 1.20×10^-01^ |

**Supplementary Table 6** Genome-wide association of damage estimates derived from different cognitive assessments. The location that was significant (P<5×10^-8^) for CR scores were non-significant for damage estimates except for *APOE*, which shows much stronger effects on damage. The purpose of this table is only to investigate overlaps of the genetic signal between CR and damage. As such, other hits for damage are not reported. The 4 symbols per gene in the direction box signify the direction of effect in each cohort in the following order: ADC1, ADC2, OMNI, GSA. The symbol ? indicates that the SNP was not available in the dataset after MAF>0.01 thresholding, - indicates a negative direction of effect (protective effect of alternate allele), and + indicates a positive direction of effect (risk effect of alternate allele).

| **Damage** | **Chr** | **Position** | **rs ID** | **Closest Gene** | **Ref** | **Alt** | **Direction** | **β (SE)** | **P-value** |
| --- | --- | --- | --- | --- | --- | --- | --- | --- | --- |
| **ANIMALS** | 8 | 2201568 | rs182140222 | *MYOM2*<>LOC101927815 | C | G | ??+- | -0.541 (0.507) | 0.287 |
| **LOGIMEM** | 19 | 44908684 | rs429358 | *APOE* | T | C | ++++ | 1.219 (0.092) | 1.18×10^-39^ |
| **NACCUDSD** | 3 | 86714172 | rs148691207 | LINC02070<>*VGLL3* | G | A | ---+ | -0.136 (0.077) | 0.077 |
| **NACCUDSD** | 6 | 170624470 | NA | LOC101929692 | AC | A | -?-? | 0.103 (0.169) | 0.542 |
| **NACCUDSD** | 19 | 44908684 | rs429358 | *APOE* | T | C | ++++ | 0.247 (0.020) | 1.77×10^-36^ |
